# Genome-wide analysis reveals novel regulators of synaptic maintenance in *Drosophila*

**DOI:** 10.1093/genetics/iyad025

**Published:** 2023-02-17

**Authors:** Jessica M Sidisky, Danielle de Paula Moreira, Meryem Okumus, Russell Caratenuto, Cassidy Drost, Bali Connors, Sarrah Hussain, Stephanie Alkhatib, Daniel T Babcock

**Affiliations:** Department of Biological Sciences, Lehigh University, 111 Research Drive, Bethlehem, PA 18015, USA; Department of Biological Sciences, Lehigh University, 111 Research Drive, Bethlehem, PA 18015, USA; Department of Biological Sciences, Lehigh University, 111 Research Drive, Bethlehem, PA 18015, USA; Department of Biological Sciences, Lehigh University, 111 Research Drive, Bethlehem, PA 18015, USA; Department of Biological Sciences, Lehigh University, 111 Research Drive, Bethlehem, PA 18015, USA; Department of Biological Sciences, Lehigh University, 111 Research Drive, Bethlehem, PA 18015, USA; Department of Biological Sciences, Lehigh University, 111 Research Drive, Bethlehem, PA 18015, USA; Department of Biological Sciences, Lehigh University, 111 Research Drive, Bethlehem, PA 18015, USA; Department of Biological Sciences, Lehigh University, 111 Research Drive, Bethlehem, PA 18015, USA

**Keywords:** *Drosophila*, synapse, neuromuscular junction, flight

## Abstract

Maintaining synaptic communication is required to preserve nervous system function as an organism ages. While much work has been accomplished to understand synapse formation and development, we understand relatively little regarding maintaining synaptic integrity throughout aging. To better understand the mechanisms responsible for maintaining synaptic structure and function, we performed an unbiased forward genetic screen to identify genes required for synapse maintenance of adult *Drosophila* neuromuscular junctions. Using flight behavior as a screening tool, we evaluated flight ability in 198 lines from the *Drosophila* Genetic Reference Panel to identify single nucleotide polymorphisms (SNPs) that are associated with a progressive loss of flight ability with age. Among the many candidate genes identified from this screen, we focus here on 10 genes with clear human homologs harboring SNPs that are most highly associated with synaptic maintenance. Functional validation of these genes using mutant alleles revealed a progressive loss of synaptic structural integrity. Tissue-specific knockdown of these genes using RNA interference (RNAi) uncovered important roles for these genes in either presynaptic motor neurons, postsynaptic muscles, or associated glial cells, highlighting the importance of each component of tripartite synapses. These results offer greater insight into the mechanisms responsible for maintaining structural and functional integrity of synapses with age.

## Introduction

Synapses are the key structures that underlie communication in the nervous system. While synaptic growth and development are well established (Turrigiano and Nelson 2004; Collins and DiAntonio 2007), relatively little is known regarding how synapses are maintained during aging. Synaptic communication is a dynamic process that requires these structures to be responsive to changes throughout all stages of life of an organism (Bishop *et al.* 2010; López-Otín *et al.* 2013). Emerging evidence has highlighted the importance of autophagy (Vijayan and Verstreken 2017) and proteostasis (Martínez *et al.* 2018) in sustaining synapses. Synaptic dysfunction is also among the earliest hallmarks of neurodegenerative diseases (Selkoe 2002; Oddo *et al.* 2003; Lodato *et al.* 2018), highlighting the central role of synaptic maintenance in sustaining neuronal function. To better understand the cellular and molecular mechanisms by which synaptic integrity is maintained, we seek to identify and characterize the genes involved in maintaining healthy synapses through aging.

One well-established model to study synaptic integrity has been the *Drosophila* neuromuscular junction (NMJ) (Jan and Jan 1976; Broadie and Bate 1993a, b). The larval NMJ is a well-characterized model that has been indispensable for our understanding of synapse growth and development (Collins and DiAntonio 2007; Harris and Littleton 2015). However, these structures only remain for a few days before being dismantled shortly after puparium formation (Liu *et al.* 2010), limiting our ability to use this model to study aging synapses over longer periods of time. In order to understand how synapses are maintained with age, one appealing model is the adult dorsal longitudinal muscle (DLM) NMJs that are required for flight behavior (Fernandes *et al.* 1991; Fernandes and VijayRaghavan 1993; Fernandes *et al.* 1996; Fernandes and Keshishian 1998; Hebbar and Fernandes 2004). These structures form tripartite synapses comprised of presynaptic motor neurons, postsynaptic muscles, and closely associated glia (Danjo *et al.* 2011). These tissues are amenable to assess both synaptic structure and function throughout the adult stage (Babcock and Ganetzky 2014; Sidisky *et al.* 2021).

One major strength of using *Drosophila* is the power of forward genetic screens to identify novel genes associated with a particular phenotype (Palladino *et al.* 2002). In addition to mutagenesis screens, using variation that exists in natural populations has emerged as a powerful tool for identifying genes associated with biological processes. The *Drosophila* Genetic Reference Panel (DGRP), for example, consists of over 200 fully sequenced isogeneic fly stocks to identify single nucleotide polymorphisms (SNPs) associated with a given trait (Mackay *et al.* 2012). The DGRP has been used in several genome-wide studies including traumatic brain injury (TBI) (Katzenberger *et al.* 2015), retinitis pigmentosa (Chow *et al.* 2016), and dopaminergic neuron viability (Davis *et al.* 2021). Additionally, DGRP studies can also use changes in locomotor behavior, such as climbing to study the decline in physical activity on lifespan (Wilson *et al.* 2020). With the strength of Genome-wide association studies studies, the DGRP could be applied to identify novel genes involved with synaptic maintenance.

In this study, we utilized the DGRP to identify novel regulators of synaptic maintenance at adult DLM NMJs in association with progressive loss of flight ability. Our screen detected variation in maintaining flight ability across DGRP lines. Here we focus primarily on the 10 most highly associated genes that have clear human homologs. We then functionally validated candidate genes that harbor SNPs associated with defects in maintaining flight ability using both mutant analysis and tissue-specific RNAi knockdown. We found that a progressive loss of flight ability is a strong indicator of defects in synaptic maintenance through quantification of gross morphology showing changes in neurite length, bouton count, and assessment of presynaptic and postsynaptic markers. Overall, our results described here identify several genes that regulate the maintenance of synaptic structure and function with age.

## Methods

### Fly stocks and husbandry

Fly stocks were maintained at 25°C on standard *Drosophila* medium. Flies used for experimental analysis were collected shortly after eclosion, separated by sex, and aged for 3 days or 21 days at 29°C.

The following fly stocks were obtained from the Bloomington *Drosophila* Stock Center: DGRP fly stocks (Mackay *et al.* 2012), *Cora^EY07598^* (#16848) (Bellen *et al.* 2004), *MSP300^MI02921^* (#43857) (Nagarkar-Jaiswal *et al.* 2015), *futsch^N94^* (#8805) (Hummel *et al.* 2000), *cv-2^225–3^* (#6342) (Conley *et al.* 2000), *serrate^D^* (#81122) (Fleming *et al.* 1990), *frq2^06131^* (#18939) (Thibault *et al.* 2004), *DOPR2^MB05108^* (#24743) (Bellen *et al.* 2011), *GMAP^EP491^* (#10130) (Bellen *et al.* 2004), *BMCP^BG02446^* (#12688) (Bellen *et al.* 2004), *pumilio^bem^* (#6782) (Stern *et al.* 1995), 10xUAS-mCD8::GFP (#32184) (Pfeiffer *et al.* 2010), Oregon-R (#5), MHC-Gal4 (#55132) (Klein *et al.* 2014), Repo-Gal4 (#7415) (Sepp *et al.* 2001), BG380-Gal4 (#80580) (Sanyal 2009), Tubulin-gal4 (Lee and Luo 1999) UAS-Dicer-2 (#24651) (Dietzl *et al.* 2007), UAS-Dicer-2 (#24650) (Dietzl *et al.* 2007), *UAS-MSP300^IR^* (#32848) (Ni *et al.* 2011), *UAS-futsch^IR^* (#40834) (Ni *et al.* 2011), *UAS-cv-2^IR^* (#37514) (Ni *et al.* 2011), *UAS-serrate^IR^* (#34700) (Perkins *et al.* 2015), *UAS-frq2^IR^* (#28711) (Ni *et al.* 2009), *UAS-DOPR2^IR^* (#65997) (Ni *et al.* 2009), *UAS-GMAP^IR^* (#64863) (Ni *et al.* 2009), and *UAS-BMCP^IR^* (#37498) (Perkins *et al.* 2015). The following fly stocks were obtained from the Vienna *Drosophila* Resource Center: *UAS-cora^IR^* (#9788) (Dietzl *et al.* 2007) and *UAS-pumilio^IR^* (#45815) (Dietzl *et al.* 2007).

### Flight behavior

Analysis of flight behavior was performed as previously described (Babcock and Ganetzky 2014). For assessment of flight behavior, flies were transferred to glass vials and released down a 90-cm tube with a removable plastic sheet coated in Tangle Trap (Tanglefoot Company). The landing average for each fly was recorded to the nearest centimeter as previously described (Sidisky *et al.* 2021). For all experiments, males and females were assayed separately. For flight experiments using the DGRP stocks, the landing height was calculated as the flight index, which is determined by subtracting the average landing height at Day 21 (D21) from the Day 3 (D3) value.

### Immunohistochemistry

DLMs were dissected as previously described (Sidisky and Babcock 2020). Briefly, preps were fixed in 4% paraformaldehyde for 30 min and washed with 1 × phosphate-buffered saline (PBS) four times. Preparations were then flash frozen using liquid nitrogen and dissected in ice-cold PBS. Samples were next treated with blocking buffer (0.1% normal goat serum, 0.2% Triton X-100) for at least 1 h at 4°C. For gross morphology assessment, tissues were stained with FITC-conjugated anti-horseradish peroxidase (HRP) 1:200 (Jackson Laboratories) for 2 h at room temperature (RT) in a dark box and then washed 4 × for 5 min each with PBS with 0.3% Triton (PBS-T). For all other experiments, tissues were blocked for at least 1 h and then treated with primary antibodies for 48 h at 4°C. After four PBS-T washes, samples were then incubated in secondary antibodies for 2 h at RT in the dark. Samples were then washed 4 × with PBS-T and mounted on glass slides with Vectashield (Vector Laboratories). The following primary antibody was used: mouse monoclonal anti-SYNORF1 1:25 (3C11, target Synapsin, Developmental Studies Hybridoma Bank) (Klagges *et al.* 1996), rabbit anti-*Drosophila* p21-activated kinase (dPak) 1:2,000 (Harden *et al.* 1996), and chicken polyclonal anti-GFP (Thermo Fisher). The following secondary antibodies were used: goat anti-mouse Alexa-488 1:200 (Invitrogen), goat anti-rabbit Alexa-568 1:200 (Invitrogen), Cy3-conjugated anti-HRP 1:500 (Jackson Laboratories), FITC-conjugated anti-HRP 1:200 (Jackson Laboratories), AF647-conjugated anti-HRP 1:25 (Jackson Laboratories), and Phalloidin 647 1:1,000 (Abcam ab176759).

### Assessment of synaptic morphology

DLM images were acquired using a 63 × oil objective (N.A. 1.4) on a Zeiss LSM 880 confocal microscope. Z-stacks of 45 slices (0.7 µm interval) were acquired at a constant depth starting from the top surface of muscle fiber D (Sidisky *et al.* 2021). For image analysis of gross morphology (Fig. 11, Supplementary Figures 1 and 7), 10 images were acquired from independent samples for each genotype. Z-stacks were transformed into maximum intensity projections using FIJI software (Schindelin *et al.* 2012). Total neurite length (µM) was measured using the Simple Neurite Tracer (SNT) plug-in to trace the HRP staining (Longair *et al.* 2011; Arshadi *et al.* 2021) and then analyzed using the Skeletonize 3D plug-in (Schindelin *et al.* 2012) for each image. Brightness and contrast were adjusted using ImageJ software (NIH) Fiji (Schindelin *et al.* 2012) and Adobe Photoshop CC2022.

**Fig. 1.**
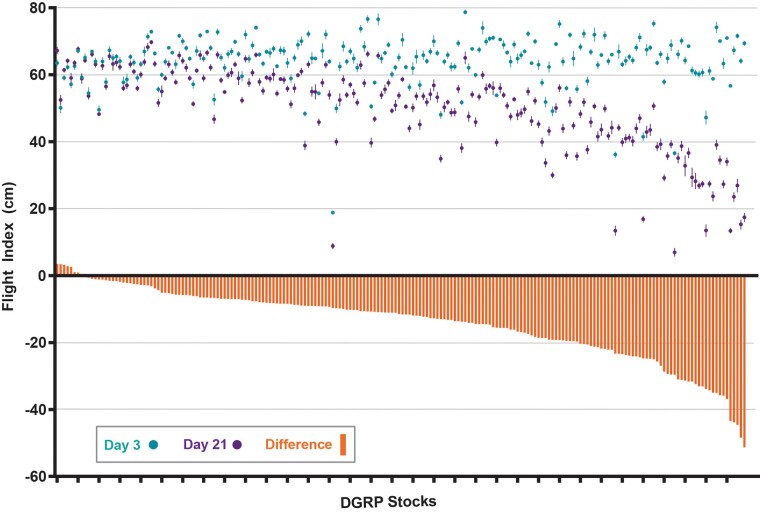
Progressive loss of flight ability varies across genetic backgrounds. Average landing heights of 198 DGRP stocks measured at Day 3 (teal dots) and Day 21 (purple dots). Orange bars represent the magnitude of difference between these values for each stock and were calculated by (D21 − D3). Error bars represent SEM. Flight ability was analyzed for each condition in triplicate.

### Quantification of bouton density and presynaptic and postsynaptic markers

DLM images for assessment of bouton density, bouton number, and presynaptic and postsynaptic markers were acquired using a 63 × objective (N.A. 1.4) on a Zeiss LSM 880 confocal microscope at a 2.5 × zoom. Confocal parameters were uniform for all images and genotypes with optimization for the synaptic markers. Z-stacks of 30 slices of muscle fiber D (1.0 µm interval) were acquired at a constant depth from the surface of the muscle fiber D. For image analysis, 12 images were acquired, 3 per sample, over 4 independent samples for an accurate assessment of synaptic markers in the anterior region of muscle fiber D for Day 3 and Day 21 females for each mutant and controls. Z-stacks were transformed into maximum intensity projections using FIJI software (Schindelin *et al.* 2012). Bouton density was calculated by first identifying synaptic boutons through the neuronal membrane marker HRP (Jan and Jan 1982) to count the boutons with FIJI's Region of Interest (ROI) tool (Schindelin *et al.* 2012). The total neurite length was accessed as described above using SNT plug-in to trace the HRP (Longair *et al.* 2011; Arshadi *et al.* 2021) and then analyzed using the Skeletonize 3D plug-in (Schindelin *et al.* 2012) for each image. Bouton density was then calculated by dividing the bouton number by total neurite length and multiplying by 1,000 µM for all samples. Brightness and contrast were adjusted using ImageJ software (NIH) Fiji (Schindelin *et al.*, 2012) and Adobe Photoshop CC2022.

To assess synaptic markers, DLM images were acquired as described above for the bouton density assessment with 12 images acquired for each group, 3 per sample, over 4 independent samples for an accurate assessment of synaptic markers in the anterior region of muscle fiber D for Day 3 and Day 21 females for each mutant and control. First boutons were identified with HRP and selected using the oval area tool in FIJI (Schindelin *et al.* 2012). The presynaptic marker Synapsin and the postsynaptic density marker dPak were assessed by counting the number of boutons positive for either marker. The synapsin- or dPak-positive boutons were calculated as a percentage out of the total number of boutons in each image. Brightness and contrast were adjusted using ImageJ software (NIH) Fiji (Schindelin *et al.* 2012) and Adobe Photoshop CC2022.

### Total RNA isolation and reverse transcription quantitative polymerase chain reaction

Total RNA was isolated from a group of 30 DLMs of 3-day-old adult female mutant flies. For the RNAi fly lines, groups of 20 whole bodies of 3-day-old adult female flies or third instar larvae were used to isolate the total RNA. Immediately after isolating each group, all tissues (DLMs/whole bodies/larvae) were flash frozen on liquid nitrogen and stored on −80°C freezer as previously described (Sidisky *et al.* 2021). Mutant lines used in these experiments are described above, and the control group was Oregon-R (also called wildtype). For RNAi experiments, F1 female flies were collected from the following crosses: (1) test groups [MHC-gal4 (muscle driver) females and males of *UAS-cora^IR^*, *UAS-MSP300^IR^*, *UAS-serrate^IR^*, *UAS-frq2^IR^*, and *UAS-pumilio^IR^* and tubulin-gal4 (ubiquitous driver) females and males of *UAS-futsch^IR^*, *UAS-CV-2^IR^*, *UAS-GMAP^IR^*, and *UAS-BMCP^IR^*] and (2) control groups (Oregon-R females and males of the RNAi lines described above). Due to technical limitations, the mRNA expression level of *DOPR2* was not assessed.

Total RNA extraction was performed with Monarch Total RNA Miniprep Kit (NEB) following tissue and leukocyte protocol as previously described (Kao *et al.* 2019). Total RNA purity and integrity was accessed on NanoDrop One Spectrophotometer (Thermo Fisher). cDNA was synthesized using qScript cDNA SuperMix (Quantabio) according to manufacturer's instruction.

Reverse transcription quantitative polymerase chain reaction (RT-qPCR) was performed using PowerUP Sybr Green Master Mix (Applied Biosystems) on an ABI7300 or a Rotor-Gene. Each assay was run in technical triplicates on ABI7300 and in technical duplicates on Rotor-Gene. Data normalization was performed against Actin5C (Dalui and Bhattacharyya 2014) and calibrated to proper control, as described on each figure. Primer efficiencies were determined, and analysis was performed with LinREgPCR (Ruijter *et al.* 2009). Primer pairs were designed using NCBI primer design tool and are listed in Supplementary Table 3.

### Statistical analysis

Data are shown as mean or mean ± SD. The statistical significance of the flight behavior, synaptic morphology, bouton density, and assessment of pre- and postsynaptic markers were analyzed using a one-way ANOVA with Tukey's post hoc comparisons. RT-qPCR data was analyzed using the Kruskal–Wallis test for more than two independent small samples and Mann–Whitney U test to compare two independent small samples. All statistical analyses were carried out using GraphPad PRISM 9 software (GraphPad Software, San Diego, CA). SNP analysis associated with the progressive loss of flight ability was generated through the DGRP pipeline (http://dgrp2.gnets.ncsu.edu/) (Huang *et al.* 2014).

## Results

### Maintenance of flight ability varies across genetic backgrounds

To identify genes required to maintain synaptic integrity, we first screened for a progressive loss of flight ability with age as an initial readout. Our previous work demonstrated a strong link between impaired flight ability and the loss of DLM synaptic integrity (Sidisky and Babcock 2020; Sidisky *et al.* 2021), suggesting that this strategy would likely uncover genes associated with synaptic maintenance. We assessed flight ability in 198 DGRP fly stocks at both Day 3 and Day 21. We chose Day 3 for our initial measurements to allow the DLM innervation pattern to stabilize following pruning that occurs during metamorphosis (Hebbar and Fernandes 2005). We used Day 21 since our previous findings revealed synaptic defects in a model of amyotrophic lateral sclerosis (ALS) at this time (Sidisky and Babcock 2020). The average landing height for each genotype was scored at both timepoints (Fig. 1, dots), and genotypes were ranked based on the difference between these values (Fig. 1, orange bars). While the majority of stocks performed well at Day 3, we found greater variability of flight performance at Day 21. Flight ability remained similar between Days 3 and 21 for some genotypes, while others showed a major reduction in flight ability with age (Fig. 1, Supplementary Table 1). Overall, these results demonstrate that maintaining flight ability with age is a phenotype that varies considerably across genetic backgrounds.

### Age-dependent flight impairments are linked to synaptic defects

To assess whether a progressive loss of flight performance is associated with impaired synaptic maintenance within DLMs, we measured NMJ morphology in DGRP stocks that ranked high or low in our initial screen. We found that NMJ integrity remained intact with age in Line 301, which showed a strong flight performance at Day 21, with total neurite length remaining consistent with age (Supplementary Fig. 1a, c, and e). In contrast, NMJ morphology in Line 91, which flew poorly at Day 21, displayed a significant decrease in neurite length from Day 3 to Day 21 (Supplementary Fig. 1b, d, and e). These data suggest that, while other factors can certainly influence flight behavior, the use of the progressive loss of flight ability is an effective strategy for identifying genes regulating synaptic maintenance.

### Identification of genes harboring SNPs associated with progressive flight defects

We identified SNPs associated with the progressive loss of flight ability by submitting the ranked order of DGRP stocks from our assay into the DGRP pipeline (http://dgrp2.gnets.ncsu.edu/) (Huang *et al.* 2014). This analysis revealed that SNPs in several genes were highly associated with a loss of flight ability (Supplementary Table 2). To begin functionally validating this data set, we initially focused on a subset of these genes that includes the 10 most highly associated genes with a loss of flight ability that also have a clear human ortholog (Table 1). To further investigate the potential roles of these genes in maintaining synaptic integrity, we acquired publicly available mutant alleles and RNAi transgenes associated with these genes for analysis.

**Table 1.** Top candidate genes with human orthologs.

Rank	Candidate gene	SNP*^a^*	*P*-value*^a^*	Human ortholog	OMIM
1	cora	2R:15117148	1.87E-07	EPB41L1	602879
2	Msp300	2L: 5154234	4.69E-07	SYNE1	608441
3	futsch	X: 1325055	5.64E-07	MAP1A	600178
4	cv-2	2R:17242555	8.13E-07	BMPER	608699
5	Serrate	3R: 23009203	1.18E-06	JAG1	601920
6	Frq2	X: 18092812	5.10E-06	NCS1	603315
7	DopR2	3R: 25457938	6.75E-06	ADRB1	109630
8	Gmap	X: 15388968	6.96E-06	TRIP11	604505
9	Bmcp	3L: 11713848	6.97E-06	SLC25A30	610793
10	pumilio	3R: 4967373	1.31E-05	PUM2	607205

Identification of genes harboring SNPs that are most significantly associated with a progressive loss of flight. This list highlights the 10 most highly associated genes that have a clear human homolog.

*S*NP with the most statistically significant association for this candidate gene.

### Mutant analysis of candidate genes reveals defects in flight behavior

**Fig. 2.**
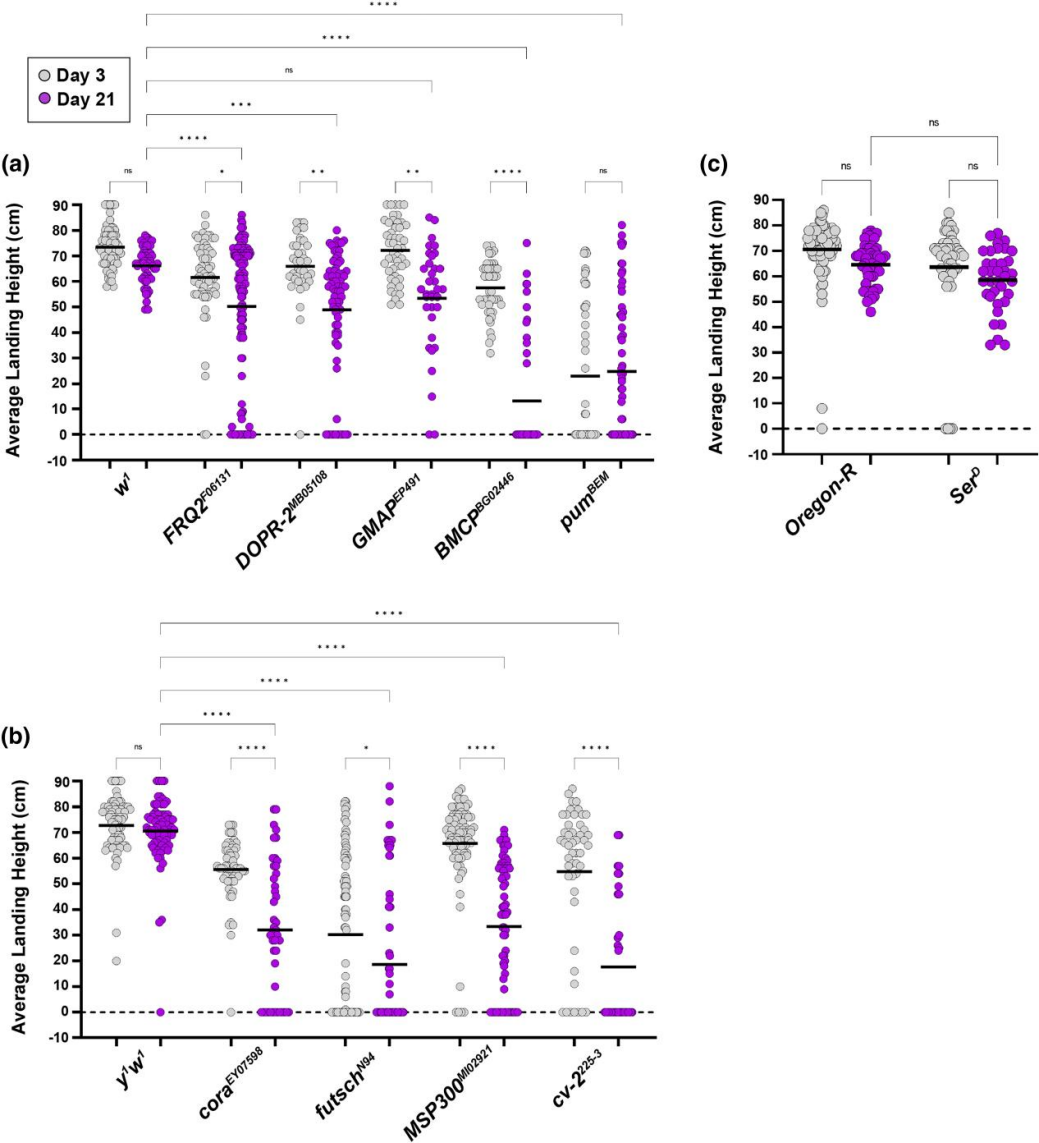
Female flight ability in mutants of candidate genes. Measurement of flight ability using females with mutant alleles for each candidate gene. Each allele was assessed in comparison to the appropriate genetic background. a) Flight ability for mutant alleles with a *white* background. b) Flight ability for mutant alleles with a *yellow*–*white* background. c) Flight ability for mutant alleles with an *Oregon-R* background. The average landing height (black bars) for each condition is assessed at Day 3 (gray dots) and Day 21 (purple dots). ****P < 0.0001; ***P < 0.001; **P < 0.01; *P < 0.05; n.s., not significant, using one-way ANOVA with Tukey's post hoc comparisons. Flight ability was analyzed for each condition in triplicate.

To functionally validate the hits from our screen, we assessed flight performance with age using mutant alleles for each candidate gene. To account for possible sex-specific differences, male and female flies were collected, raised, and assessed separately. Although we did not encounter any sex-specific differences in flight performance for DGRP fly stocks, we did uncover some differences with a few mutant alleles (Fig. 2 and Supplementary Fig. 2). Because of this, we analyzed male and female flight performance separately. For each allele, flight ability was measured at both Day 3 and Day 21, and transcript levels were assessed for changes in gene expression (Supplementary Fig. 3).

In our mutant analysis of female flies, we found that mutations in 9 of the 10 candidate genes showed a significant flight defect in comparison to wildtype controls from their genetic background (Fig. 2). The only exception was the allele *Ser^D^*, which did not differ significantly from the *Oregon-R* control. Mutations in eight of these genes displayed a progressive, age-dependent loss of flight ability [*cora*, *futsch*, *msp300*, *Crossveinless 2* (*cv-2*), *FRQ2*, *DopR2*, *GMAP*, and *BMCP*], while the *pumilio* showed severe flight defects at both Day 3 and Day 21. Interestingly, a significant decrease in the transcript level was only detected in Golgi microtubule-associated protein (GMAP) and BMCP alleles (Supplementary Fig. 3). This suggests that some of the flight defects are due to factors beyond a simple reduction in the gene expression level.

When performing mutant analysis using male flies, we found that mutations in 8 of the 10 candidate genes displayed a significant flight defect compared to wildtype controls from their genetic background (Supplementary Fig. 2). Among these eight mutant alleles, six showed a progressive, age-dependent decline in flight performance (*cora*, *msp300*, *cv-2*, *serrate*, *frq2*, *BMCP*), while the other two (*futsch*, *pumilio*) showed a significant flight defect even at Day 3. Neither of the mutant alleles for the two remaining genes (*GMAP* and *DOPR2*) showed a flight defect (Supplementary Fig. 2). It is currently unclear why sex-specific differences in flight performance exist specifically with these two alleles, but this could help determine the mechanism by which these genes help maintain flight performance with age. Altogether, these results align with the initial findings from our DGRP screen and highlight the roles of these genes in maintaining flight performance with age.

### Mutations in candidate genes disrupt NMJ morphology

**Fig. 3.**
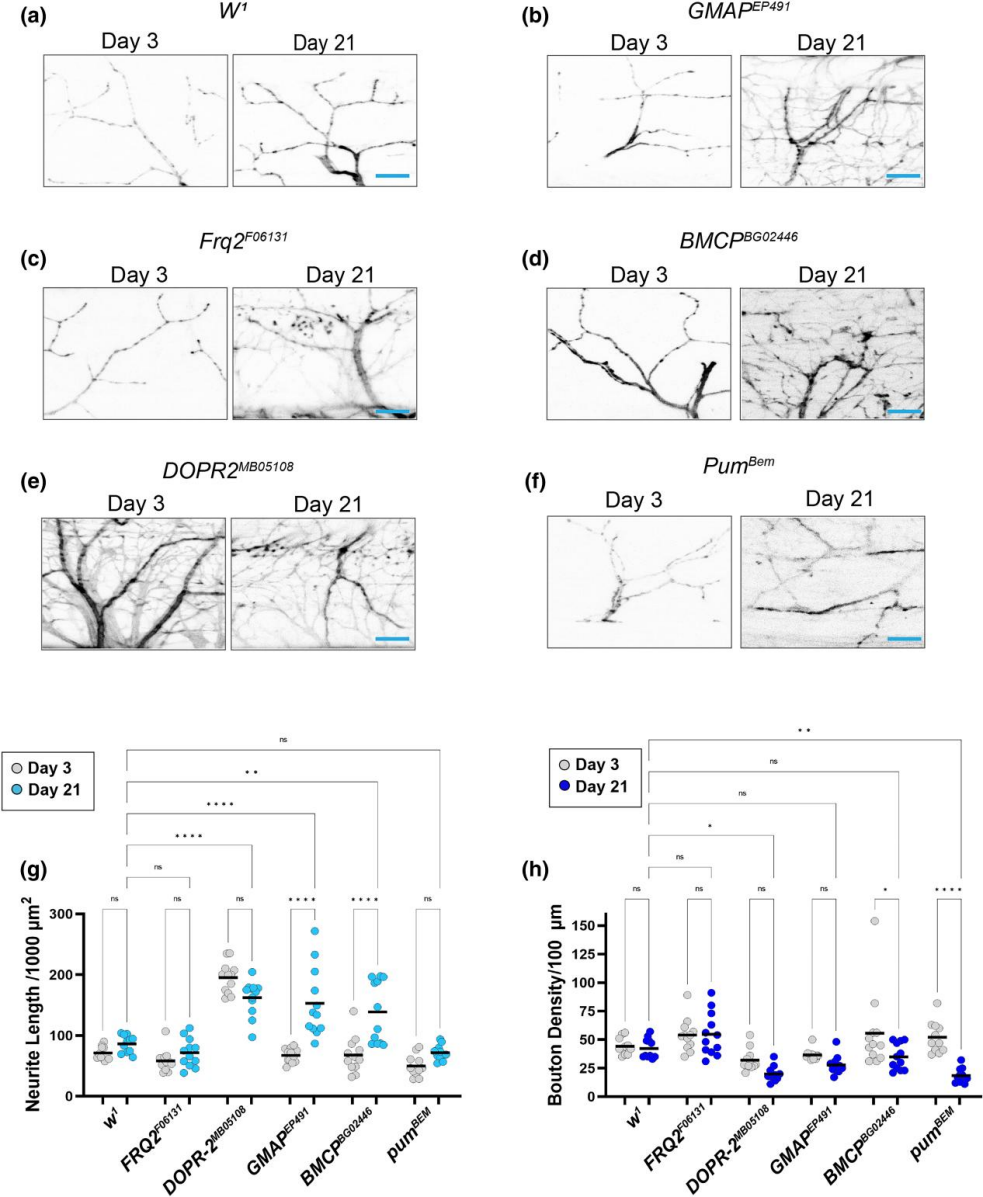
Synaptic morphology in candidate gene mutations in a *white* background. a–f) Confocal images of *white* mutants as well as alleles of candidate genes in this background stained with 647-conjugated HRP (black) at 63 × magnification. g) Quantification of total neurite length for each allele at Day 3 (gray dots) and Day 21 (blue dots). h) Quantification of bouton density for each allele at Day 3 (gray dots) and Day 21 (blue dots). Sample size = *n* of 10 for each timepoint and genotype. ****P < 0.0001; **P < 0.01; *P < 0.05; n.s., not significant, using a one-way ANOVA with Tukey's post hoc comparisons. The scale bar is 10 µm for each pair of images.

Since mutant alleles for each of the candidate genes showed a significant loss of flight performance, we next tested whether this functional deficit is accompanied by a loss of structural integrity at DLM synapses. Since 5 of the 10 alleles are in a *white* mutant background, we first examined these alleles in comparison to their wildtype control (Fig. 3). We measured total neurite length at Day 3 and Day 21 in *GMAP*, *frq2*, *BMCP*, *DopR2*, and *pum* mutants, which each showed a progressive loss of flight performance in females. We found that total neurite length in *white* mutants did not change significantly with age (Fig. 3a and g). The values for *frq2* and *pum* mutants similarly remained unchanged (Fig. 3c, f, and g). Surprisingly, we detected increased neurite length at Day 21 in DopR2, GMAP, and BMCP mutants compared to Day 3. This suggests that alterations to neurite length alone do not account for the loss of flight performance.

In addition to neurite length, we also measured bouton density in each of these mutant alleles to assess synaptic morphology (Fig. 3h). We found a significant decrease in bouton density in *DopR2*, *BMCP*, and *pum* alleles, suggesting that changes to boutons more closely align with flight defects than neurite length itself.

**Fig. 4.**
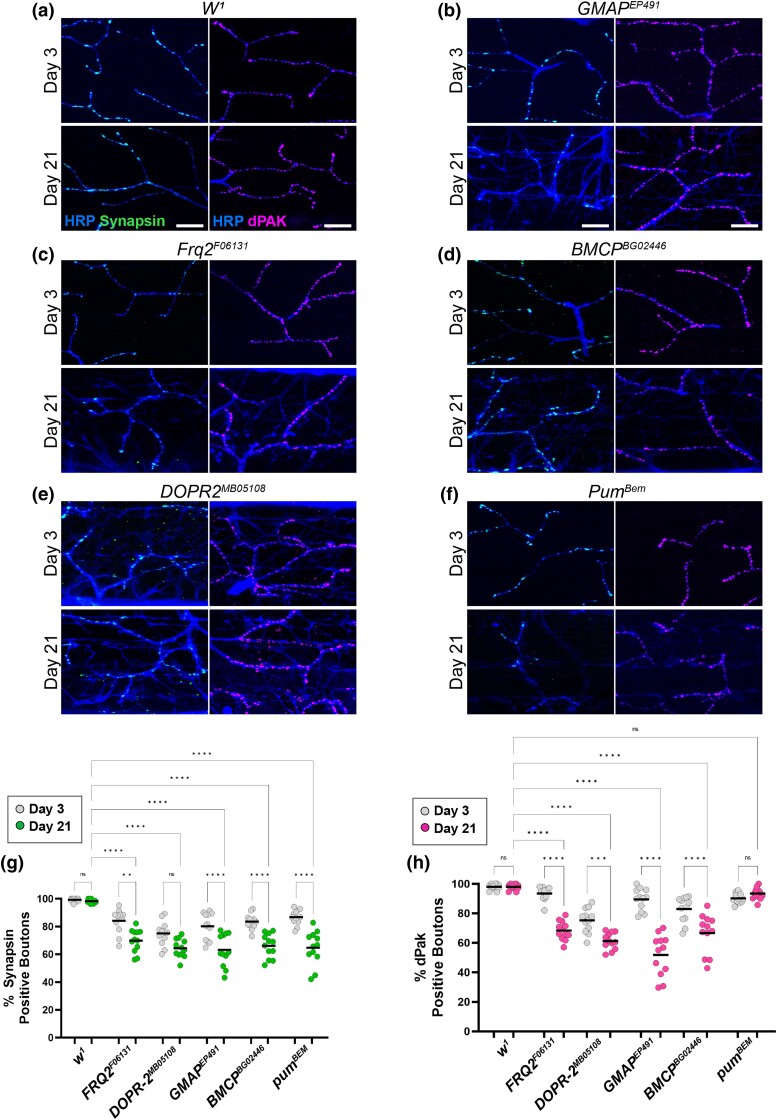
Presynaptic and postsynaptic defects in candidate gene mutations in a *white* background. a–f) Confocal images of *white* mutants as well as alleles of candidate genes in this background stained with 647-conjugated HRP (blue) at 63 × magnification. Synapses are labeled using the presynaptic marker synapsin (green) or dPak (magenta) at both Day 3 and Day 21. g) Quantification of the percentage of boutons that are synapsin-positive at Day 3 (gray dots) and Day 21 (green dots). h) Quantification of the percentage of boutons that are dPak-positive at Day 3 (gray dots) and Day 21 (magenta dots). Sample size = *n* of 12 for each timepoint and genotype. ****P < 0.0001; ***P < 0.001; **P < 0.01; *P < 0.05; n.s., not significant, using a one-way ANOVA with Tukey's post hoc comparisons. The scale bars are 10 µm for each pair of images.

To determine whether more subtle changes to synaptic structure beyond gross morphology occur in these mutant alleles, we also analyzed presynaptic and postsynaptic markers in these conditions. In each of these mutant alleles, we measured the percentage of synaptic boutons that stained positive for the presynaptic marker synapsin as well as the postsynaptic marker dPak at both Day 3 and Day 21. All five of the mutant alleles in this genetic background showed a progressive loss of synapsin-positive boutons by Day 21 compared to controls (Fig. 4). Similarly, we found a significant decrease in dPak-positive boutons by Day 21 in four of the five alleles, with the *pum* allele being the only exception. Together, these results demonstrate that defects with synaptic proteins correlate to age-dependent defects in synaptic function much more closely than changes to gross morphology.

**Fig. 5.**
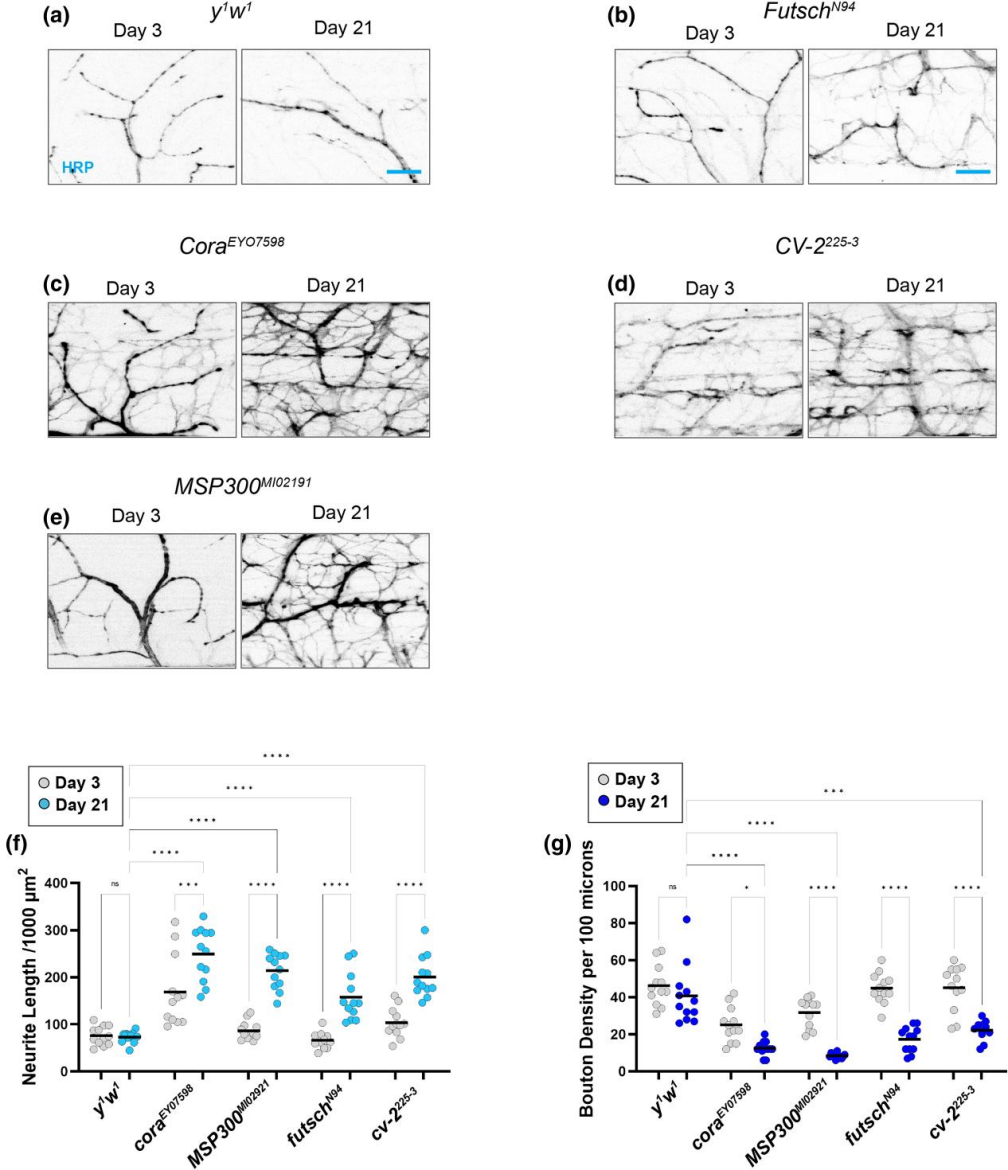
Synaptic morphology in candidate gene mutations in a *yellow*–*white* background. a–e) Confocal images of *yellow*–*white* mutants as well as alleles of candidate genes in this background stained with 647-conjugated HRP (black) at 63 × magnification. f) Quantification of total neurite length for each allele at Day 3 (gray dots) and Day 21 (blue dots). g) Quantification of bouton density for each allele at Day 3 (gray dots) and Day 21 (blue dots). Sample size = *n* of 12 for each timepoint and genotype. ****P < 0.0001; ***P < 0.001; **P < 0.01; *P < 0.05; n.s., not significant, using a one-way ANOVA with Tukey's post hoc comparisons. The scale bar is 10 µm for each pair of images.

We next performed the same analysis using mutant alleles for candidate genes generated in a separate *yellow*–*white* background. When measuring progressive changes to neurite length, we similarly found a progressive increase in neurite length compared to *yw* controls with mutant alleles of *futsch*, *cora*, *cv-2*, and *msp300* (Fig. 5a–f). However, there was also a progressive loss of bouton density in each of these mutants (Fig. 5g). This may suggest that the increased neurite length may not be completely functionally intact.

**Fig. 6.**
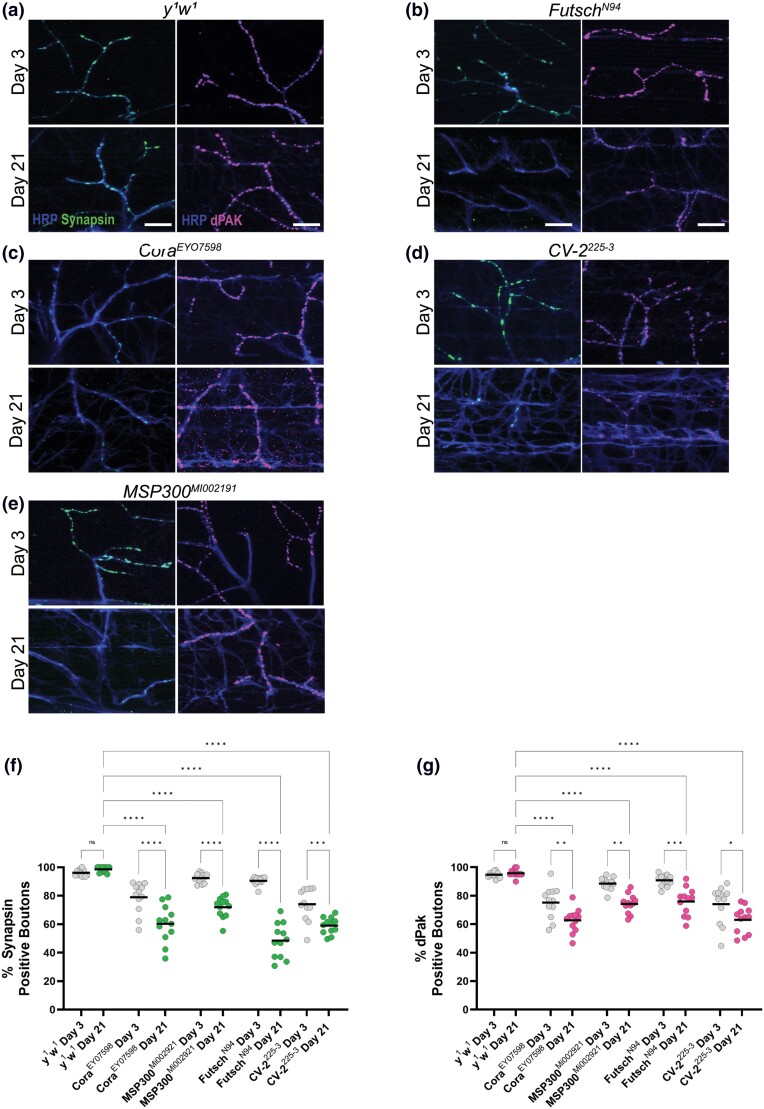
Presynaptic and postsynaptic defects in candidate gene mutations in a *yellow*–*white* background. a–e) Confocal images of *white* mutants as well as alleles of candidate genes in this background stained with 647-conjugated HRP (blue) at 63 × magnification. Synapses are labeled using the presynaptic marker synapsin (green) or dPak (magenta) at both Day 3 and Day 21. f) Quantification of the percentage of boutons that are synapsin-positive at Day 3 (gray dots) and Day 21 (green dots). g) Quantification of the percentage of boutons that are dPak-positive at Day 3 (gray dots) and Day 21 (magenta dots). Sample size = *n* of 12 for each timepoint and genotype. ****P < 0.0001; ***P < 0.001; **P < 0.01; *P < 0.05; n.s., not significant, using a one-way ANOVA with Tukey's Post hoc comparisons. The scale bars are 10 µm for each pair of images.

We also analyzed presynaptic and postsynaptic markers in these mutant alleles with age. Similar to the other mutant alleles, we found a progressive loss of both synapsin-positive and dPak-positive boutons in mutant alleles of *futsch*, *cora*, *cv-2*, and *msp300* (Fig. 6). Overall, these mutant alleles displayed remarkably similar phenotypes compared to those in other genetic backgrounds.

**Fig. 7.**
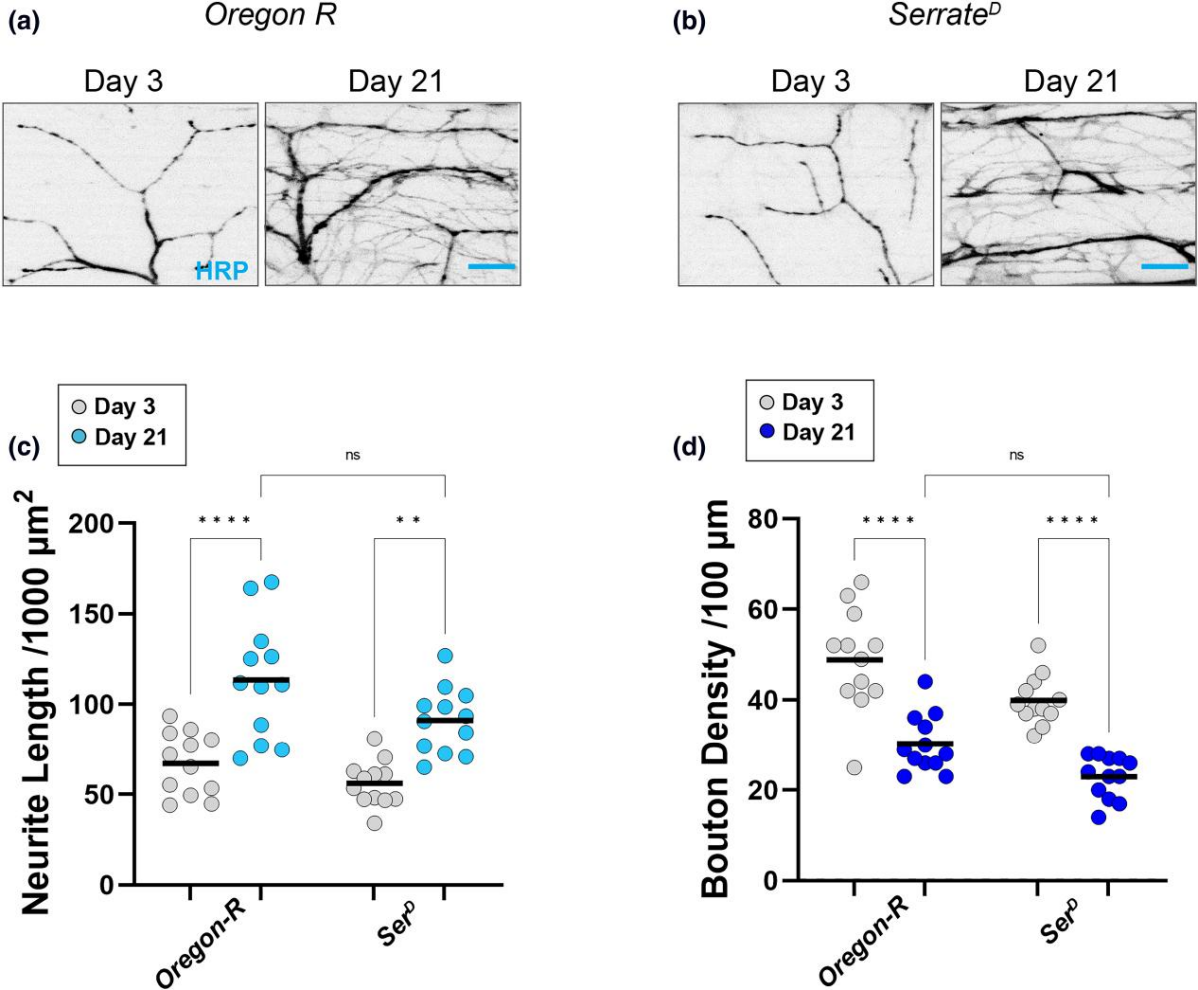
Synaptic morphology in candidate gene mutations in an *Oregon-R* background. a, b) Confocal images of *Oregon-R* mutants as well as the *serrate* allele in this background stained with 647-conjugated HRP (black) at 63 × magnification. c) Quantification of total neurite length for each allele at Day 3 (gray dots) and Day 21 (blue dots). d) Quantification of bouton density for each allele at Day 3 (gray dots) and Day 21 (blue dots). Sample size = *n* of 12 for each timepoint and genotype. ****P < 0.0001; **P < 0.01; n.s., not significant, using a one-way ANOVA with Tukey's post hoc comparisons. The scale bar is 10 µm for each pair of images.

Finally, we measured NMJ gross morphology and synaptic markers in an allele of *serrate* in comparison to *Oregon-R* controls. When assessing gross morphology in *serrate* mutants, we again observed a progressive increase in total neurite length and a progressive decrease in bouton density (Fig. 7). However, we found similar phenotypes in the *Oregon-R* controls, revealing that the genetic background is responsible, at least to some extent, for the alterations to synaptic gross morphology in these mutants.

**Fig. 8.**
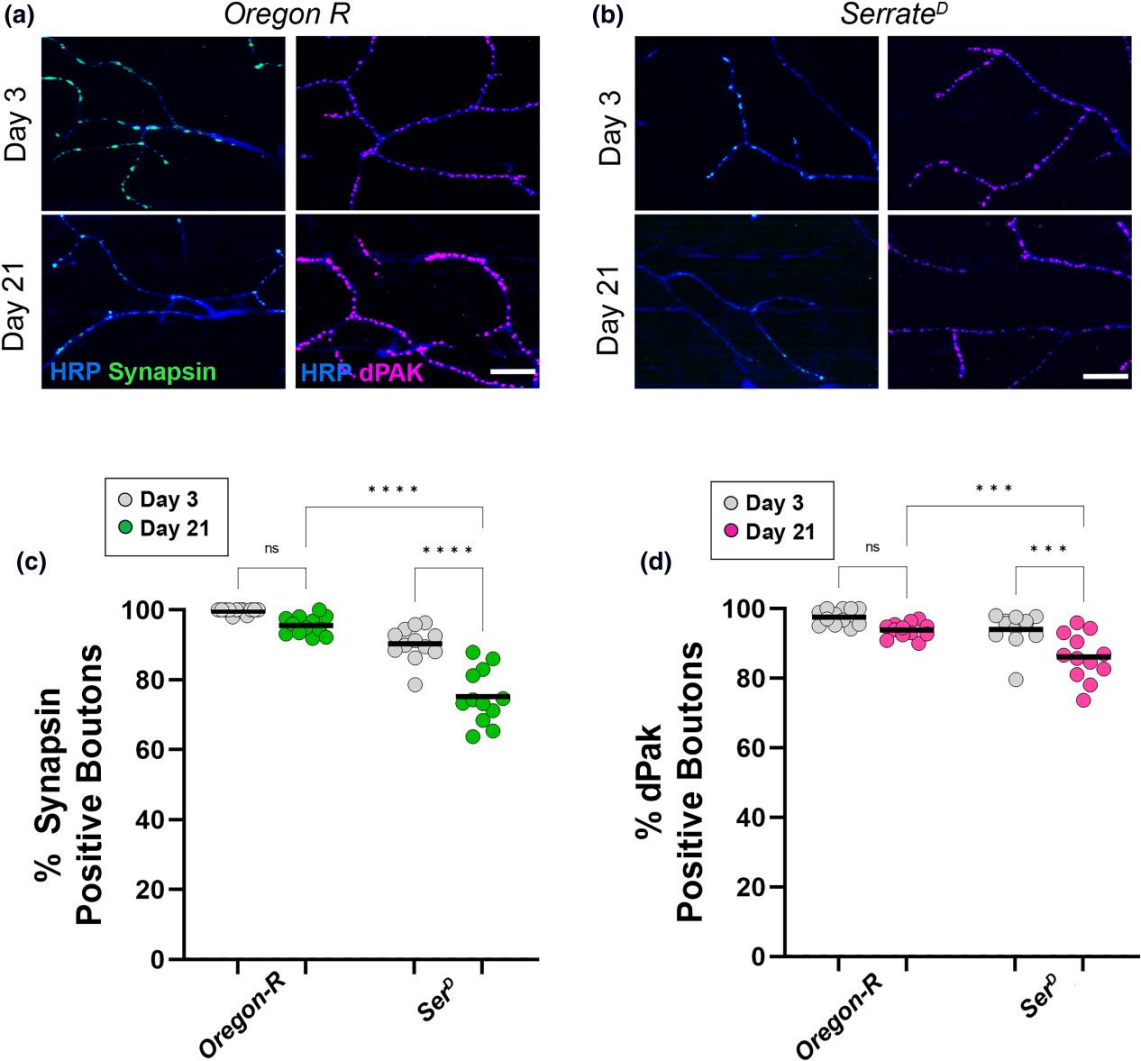
Presynaptic and postsynaptic defects in candidate gene mutations in an *Oregon-R* background. a, b) Confocal images of *Oregon-R* mutants as well as the *serrate* allele in this background stained with 647-conjugated HRP (blue) at 63 × magnification. Synapses are labeled using the presynaptic marker synapsin (green) or dPak (magenta) at both Day 3 and Day 21. c) Quantification of the percentage of boutons that are synapsin-positive at Day 3 (gray dots) and Day 21 (green dots). d) Quantification of the percentage of boutons that are dPak-positive at Day 3 (gray dots) and Day 21 (magenta dots). Sample size = *n* of 12 for each timepoint and genotype. ****P < 0.0001; ***P < 0.001; n.s., not significant, using a one-way ANOVA with Tukey's post hoc comparisons. The scale bars are 10 µm for each pair of images.

While the changes to gross morphology in *serrate* mutants appear to depend on genetic background, we found very different results using synaptic markers in these mutants. We observed a progressive loss of both synapsin-positive and dPak-positive boutons in *serrate* mutants compared to controls (Fig. 8). Altogether, these results suggest that mutations in most of the candidate genes from our screen display an age-dependent loss of flight ability, accompanied by an increase in neurite length, decrease in bouton density, and progressive loss of both presynaptic and postsynaptic markers.

### Tissue-specific knockdown of candidate genes highlights the roles of neurons, muscles, and glia in synaptic maintenance

**Fig. 9.**
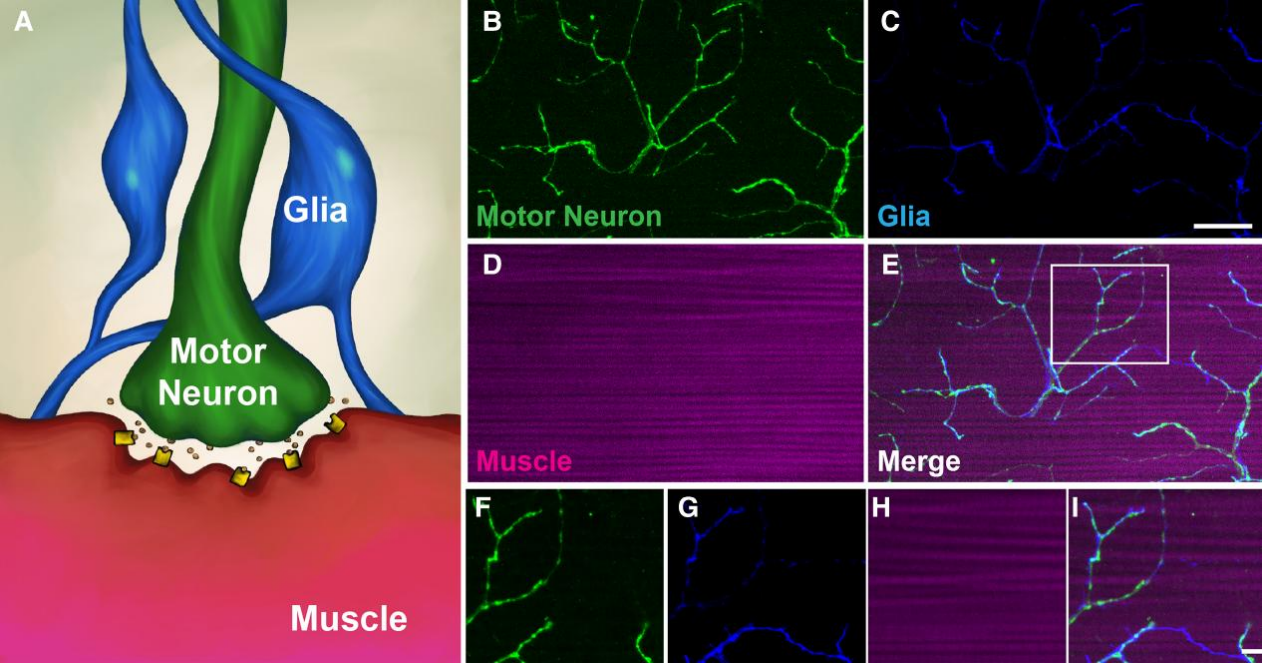
Structure of the DLM tripartite synapse. a) Illustration of the tripartite DLM synapse, highlighting motor neurons that innervate the DLMs, as well as glial cells that are tightly associated with these structures. b–e) Confocal analysis of motor neurons (green), glial cells (blue), and muscles (magenta) to demonstrate structural layout of DLM synapses. Glial cells are labeled by expressing a membrane-bound GFP using Repo-Gal4 and false-colored blue. Motor neurons are labeled using Cy3-HRP and colored green. Muscles are labeled using 647-phalloidin and colored magenta. f–i) Higher magnification of synaptic structures as seen within boxed region of e. Scale bar in c is 20 µm for b–e. Scale bar in i is 10 µm for f–i.

The NMJs located within the DLMs are tripartite synapses (Danjo *et al.* 2011) that consist of presynaptic motor neurons, postsynaptic muscles, and closely associated glial cells (Fig. 9a). This arrangement allows for the analysis of each of these structures (Fig. 9b–i).

**Fig. 10.**
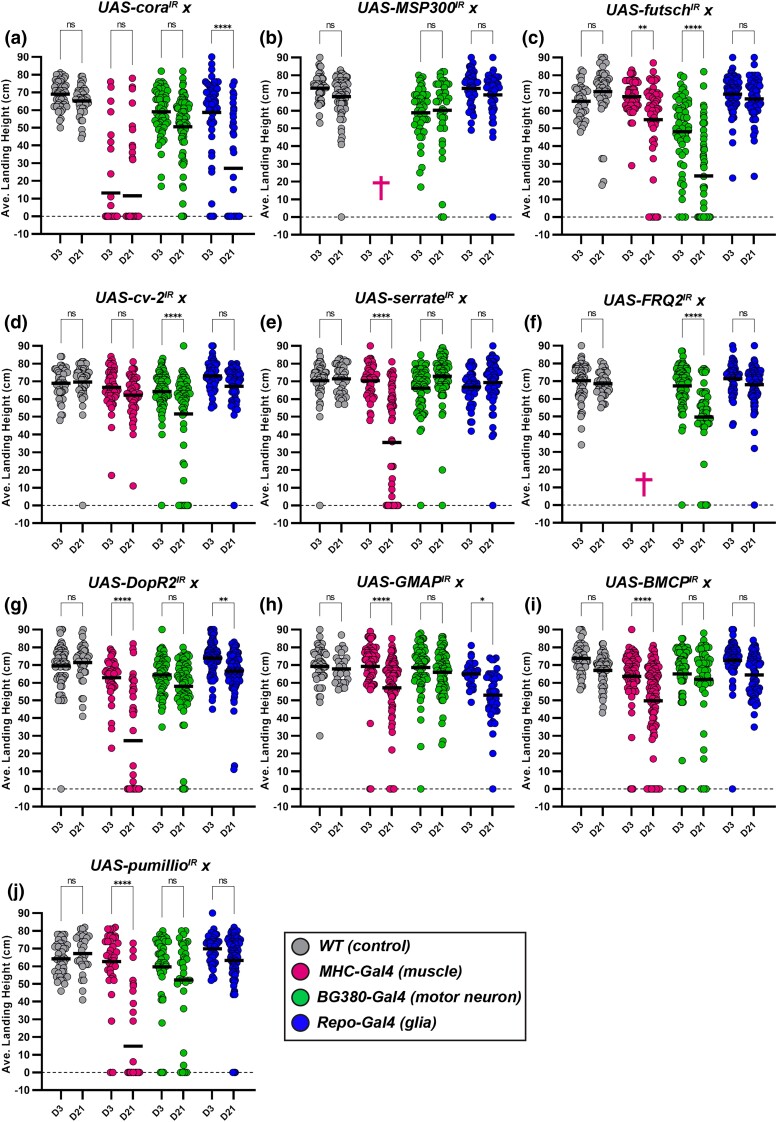
a–j) Female flight ability with RNAi knockdown of candidate genes. Measurement of flight ability in females using tissue-specific knockdown of each candidate gene. Each gene was knocked down in muscles (magenta), motor neurons (green), and glia (blue) and also compared to controls (gray). The average landing height (black bars) for each condition is assessed at Day 3 and Day 21. All dots represent individual data points. ****P < 0.0001; ***P < 0.001; **P < 0.01; *P < 0.05; n.s., not significant, using one-way ANOVA with Tukey's post hoc comparisons. Flight ability was analyzed for each condition in triplicate.

To uncover the tissue(s) in which our candidate genes are required to maintain synaptic integrity, we selectively knocked each of these genes down in either muscles (*MHC-Gal4*), motor neurons (*BG380-Gal4*), or glia (*Repo-Gal4*). Tissue-specific knockdown of candidate genes in female flies (Fig. 10) revealed roles for each component of the tripartite synapse in maintaining synaptic integrity.

Upon knockdown in the muscle, nearly every candidate displayed a strong phenotype. Knockdown of *MSP300* or f*rq2* was lethal (Fig. 10b and f and Supplementary Table 4), while knockdown of *cora* showed a strong flight defect even at an early timepoint (Fig. 10a). Measurement of transcript levels confirmed that the majority of the candidate genes had a significant decrease in expression upon RNAi-mediated knockdown (Supplementary Fig. 6). The remaining genes, with the exception of *cv-2*, showed a progressive, age-dependent loss of flight ability, demonstrating the need for most of these candidate genes within postsynaptic muscles.

We also knocked down expression of each candidate gene in presynaptic motor neurons. We found that knocking down 3 of the 10 candidate genes resulted in a progressive loss of flight performance, as seen upon knockdown of *futsch*, *cv-2*, or f*rq2* (Fig. 10c, d, and f).

Finally, we examined glial-specific knockdown of each candidate gene in maintaining flight performance. We found small but significant effects upon knockdown of either *DopR2* or *Gmap* in glia (Fig. 10g–h), while the most striking progressive loss of flight ability was found by knocking down *cora* (Fig. 10a).

We also analyzed flight performance in male flies using the same genotypes. The phenotypes found in male flies largely recapitulated the results found using females (Supplementary Fig. 4), with a few notable exceptions. Glial-specific knockdown of *cora*, for example, was lethal in male flies (Supplementary Fig. 4a). Additionally, muscle-specific knockdown of *cv-2* resulted in progressive loss of flight ability (Supplementary Fig. 4d). Finally, motor neuron-specific knockdown of *cora*, *msp300*, *serrate*, and *pumilio* showed a much stronger flight defect in male flies compared with females (Supplementary Fig. 4a, b, e, and j). We also measured flight performance using each Gal4 driver alone (Supplementary Fig. 5) as additional controls and only observed a small yet significant change in flight behavior using *BG380-Gal4*. We did not observe any additional flight defects in these controls, suggesting that the flight defects found are primarily due to the specific knockdown of the candidate genes.

Altogether, these results highlight the need for our candidate genes to maintain flight performance through their expression in various synaptic tissues. Muscle, motor neurons, and glia each had several genes that were required within these tissues. Interestingly, the majority of our candidate genes are required in multiple tissues, suggesting shared mechanisms of maintenance in muscles, motor neurons, and glia.

### RNAi-mediated knockdown of candidate genes disrupts NMJ morphology

To assess whether tissue-specific knockdown of candidate genes impairs synaptic integrity at DLM synapses, we measured gross morphology of NMJs upon knockdown of these genes knocked down in various tissues. We chose one example of a condition resulting in progressive flight defects in each tissue: muscle, motor neuron, and glia.

**Fig. 11.**
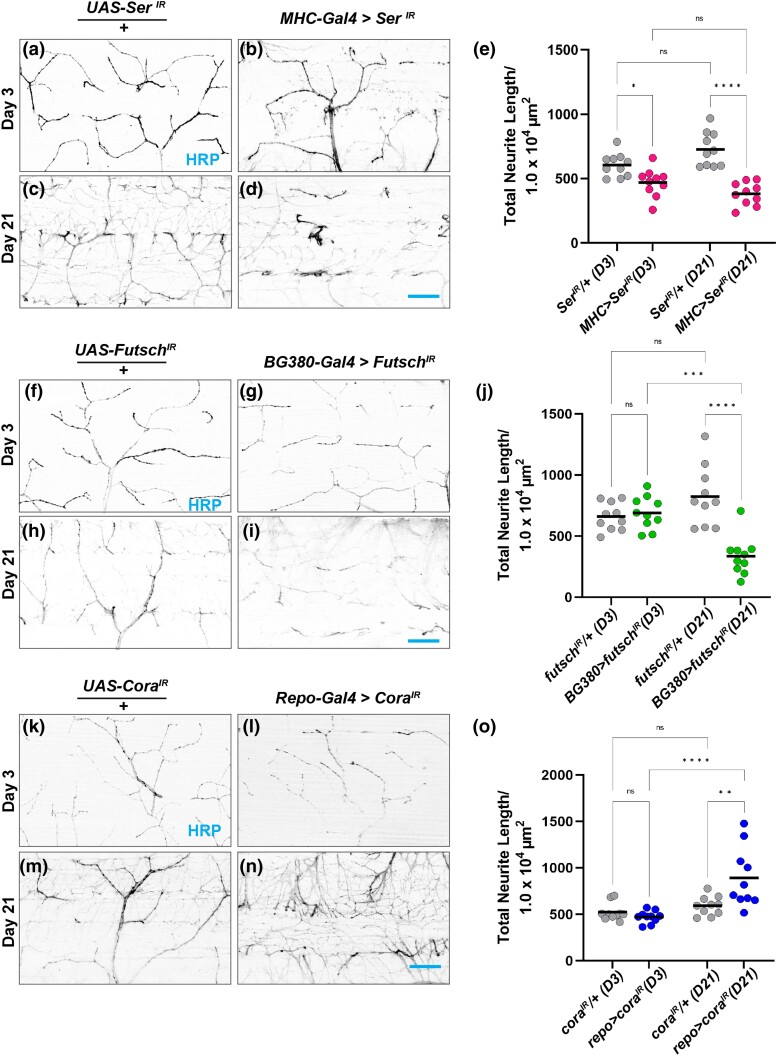
Synaptic defects with RNAi knockdown of candidate genes. a–d) Confocal images of *ser* RNAi knockdown in muscle tissue using MHC-Gal4 stained with FITC-conjugated HRP (black) at 63 × magnification. UAS-*ser* ^IR^ crossed to *Oregon-R* serves as the control. e) Quantification of total neurite length of *ser* RNAi muscle knockdown (µm). f–i) Confocal images of motor neuron knockdown of *futsch* using BG380-Gal4 and controls at Day 3 and Day 21. j) Quantification of total neurite length of *futsch* RNAi motor neuron knockdown. k–n) Representative images of *cora* glia RNAi knockdown using Repo-Gal4 and controls. o) Quantification of total neurite length of *cora* glia RNAi knockdown. Sample size = *n* of 10 for each timepoint and genotype. ****P < 0.0001; ***P < 0.001; **P < 0.01; *P < 0.05 n.s., not significant, using a one-way ANOVA with Tukey's post hoc comparisons. The scale bar in d, i, and n is 20 µm.

We first accessed NMJ morphology upon muscle-specific knockdown of *serrate* in postsynaptic muscle tissue. At Day 3, *serrate* knockdown showed early signs of impairment with a decrease in neurite length compared to the control (Fig. 11a, b, and e). By Day 21, the *serrate* knockdown showed a progressive loss of neurite length compared with the age-matched controls (Fig. 11c–e). We also confirmed that the use of the Gal4 drivers alone did not produce a change in neurite length (Supplementary Fig. 7). These results demonstrate a role for *serrate* in retaining synaptic integrity through postsynaptic muscle tissue.

We next assessed NMJ morphology upon knockdown of *futsch* in motor neurons. At Day 3, motor neuron knockdown of *futsch* resulted in a total neurite length comparable to the control (Fig. 11f, g, and j). However, by Day 21, *futsch* knockdown displayed a significant decrease in total neurite length when compared to Day 3 (Fig. 11g and j) and age-matched controls (Fig. 11h–j). These data demonstrate the role of *futsch* in maintaining synaptic structure by acting in presynaptic motor neurons.

To investigate the role of glia in preserving synaptic integrity, we assessed how knockdown of *cora* in glia impacts synaptic morphology (Fig. 11k–o). At Day 3, we observed no change in neurite length between glia knockdown of *cora* and the controls (Fig. 11k, l, and o). In contrast, by Day 21, *cora* knockdown in glia presented a significant increase in neurite length compared to the Day 21 control and the Day 3 knockdown (Fig. 11l–o). Both the flight defect in glia knockdown of *cora* (Fig. 10a) and the increase in neurite length suggests that *cora* may have a role in regulating neurite growth in glia to sustain synaptic integrity. Together, these results support the tissue-specific roles of each gene as regulators of synaptic maintenance.

## Discussion

Our results highlight genes with a wide variety of functions that mediate synaptic maintenance with age. Mutations in the vast majority of candidate genes recapitulated the results uncovered through SNP analysis using the DGRP, and tissue-specific knockdown of these genes revealed critical roles for these genes in various tissues that comprise the tripartite synapse.

One striking result uncovered from this screen was the finding that the majority of these candidate genes have roles in multiple tissues to maintain synaptic integrity. This highlights the important features of motor neurons, muscles, and glia needed to keep these complex structures maintained with age.

Although further studies will be needed to identify the specific mechanisms by which these genes are required to maintain synaptic integrity, we briefly highlight the known functions of these genes to determine how they may be involved in this process.

### Muscle development and function

As a ligand for the Notch receptor, serrate is most commonly associated with wing morphogenesis (Speicher *et al.* 1994). However, the flight defects and disruption of synaptic morphology seen here indicate a specific role for serrate within muscle cells. Previous work has demonstrated a role for serrate in the development of the indirect flight muscles. Specifically, serrate is required for proliferation of adult muscle progenitor (AMP) cells, from which the indirect flight muscles develop (Gunage *et al.* 2014). Our results showing a progressive, age-dependent loss of synaptic maintenance suggest that serrate is also required in muscles after their development.

Notch signaling has also been shown to affect synapse morphology in the olfactory circuit in *Drosophila*. Activation of the Notch ligand Delta in postsynaptic projection neurons acts back on presynaptic olfactory receptor neurons for canonical Notch signaling, thus restricting the overall architecture of glomeruli (Kidd and Lieber 2016). Since muscles are the postsynaptic cells of NMJs, our results suggest that serrate could regulate synaptic morphology via a similar mechanism. Serrate was also identified as a modifier of TDP-43-mediated toxicity in a *Drosophila* model of ALS (Zhan *et al.* 2013), further highlighting the role of serrate in maintaining neuromuscular integrity.

Human Jagged-1 (JAG1) likewise acts as a Notch ligand, with mutations identified in patients afflicted by the hereditary axonal neuropathy Charcot–Marie–Tooth disease type 2 (CMT2) (Sullivan *et al.* 2020). With human JAG1 being required to maintain peripheral nerve integrity, it will be interesting to determine how muscle-specific knockdown of serrate maintains synaptic integrity in our *Drosophila* model.

We also found that muscle-specific knockdown of BMCP impairs synaptic maintenance. BMCP, which acts as a mitochondrial carrier protein, is proposed to regulate energy metabolism. Loss of function mutations in BMCP result in starvation sensitivity, decreased fertility, and reduced weight gain on a high-calorie diet, suggesting a role in maintaining metabolic homeostasis (Sánchez-Blanco *et al.* 2006). In contrast, overexpression of BMCP rescued locomotor impairments and the shortened lifespan resulting from expression of mutant huntingtin in glial cells (Besson *et al.* 2010). Our results showing a muscle-specific requirement of BMCP for maintaining synaptic integrity could be due to the fact that the indirect flight muscles use an incredible amount of energy (Wigglesworth 1949) and include an abundance of mitochondria (Sohal 1975).

The mammalian homolog solute carrier family 25 (SLC25A30) is associated with the transport of solutes across the inner mitochondrial membrane. In mice, SLC25A30 expression is most abundant in the kidney, where it is proposed to respond to increased mitochondrial metabolism (Haguenauer *et al.* 2005). Our results in the current study suggest that mitochondrial activity in DLMs plays a crucial role in maintaining synaptic integrity.

### Synaptic development

Coracle physically interacts with postsynaptic glutamate receptors (GluRs) and is required for proper synaptic localization and anchoring of receptors with GluRIIa subunits. Loss of function mutations in *coracle* results in decreased GluRIIa localization at both embryonic and larval NMJs (Chen *et al.* 2005). Our results are also consistent with a postsynaptic role in muscles, as both structural and functional integrity of DLM synapses are impaired following muscle-specific knockdown of *coracle*. It is possible that failure to maintain proper GluR localization leads to the morphological synaptic defects presented in this study.

Interestingly, we also found synaptic impairment when *coracle* is knocked down in glial cells. Previous work has demonstrated that coracle is required for the formation of septate junctions (Lamb *et al.* 1998). Additionally, loss of *coracle* function disrupts the integrity of the blood–brain barrier in *Drosophila* embryos (Stork *et al.* 2008), which is primarily formed by glial cells. Finally, knockdown of coracle in epidermal cells promotes dendrite overgrowth in sensory neurons (Tenenbaum *et al.* 2017). It is certainly possible that coracle also acts to limit synaptic growth by acting in glia that is closely associated with NMJs.

The human homolog of Cora is similarly involved in regulating GluR activity. Specifically, erythrocyte membrane protein band 4.1-like 1 (EPB41L1) regulates the insertion of AMPA receptors at glutamatergic synapses (Lin *et al.* 2009). Mutations in EPB41L1 disrupt glutamatergic systems in nonsyndromic intellectual disability (Hamdan *et al.* 2011), highlighting the role of regulating postsynaptic receptors in neuronal maintenance. Additionally, knocking out protein 4.1B, another member of the family of proteins 4.1, in mice causes peripheral nerve abnormalities and decreased levels of Caspr2, which has a *Drosophila* ortholog dNrxIV, another component of the septate junction (Buttermore *et al.* 2011; Cifuentes-Diaz *et al.* 2011; Einheber *et al.* 2013). Our results together with these previous data show that the family of proteins 4.1 is essential for the peripheral axon–glia interaction.

Frequenin 2 (Frq2) is a calcium-binding protein that regulates neurotransmitter release as well as bouton formation. Knockdown of *frq2* in motor neurons resulted in reduced quantal content, increased bouton number, and fewer active zones per bouton at larval NMJs, while the opposite phenotypes were observed in gain-of-function conditions (Romero-Pozuelo *et al.* 2007). Our results confirm a role for Frq2 in adult motor neurons but also show defects upon muscle-specific knockdown. Further studies will be needed to better understand how loss of Frq2 impacts the structure and function of both pre- and postsynaptic tissues.

Human neuronal calcium sensor 1 (NCS1) functions as a calcium ion sensor to modulate synaptic activity and secretion (Bourne *et al.* 2001). Evidence has also shown that the calcium-binding activity of NCS1 specifically modulates A-type K^+^ currents (Nakamura *et al.* 2001).

Futsch is a microtubule-associated protein with a well-established role in both neurite and synapse development (Hummel *et al.* 2000; Roos *et al.* 2000). It is thus unsurprising that disrupting Futsch function can dismantle synaptic structure and function. Human microtubule-associated protein 1a (MAP1A) similarly acts to stabilize microtubules and is found primarily within the central nervous system (Fink *et al.* 1996).

MSP-300, also referred to as *Drosophila* nesprin-1, is responsible for the proper positioning of organelles such as mitochondria and nuclei within muscle cells (Elhanany-Tamir *et al.* 2012). Additionally, MSP-300 has been shown to regulate synapse maturation by localizing specific mRNAs (Packard *et al.* 2015) as well as GluRs (Morel *et al.* 2014) to postsynaptic sites. Our findings are consistent with a role of MSP-300 in postsynaptic muscle cells, as muscle-specific knockdown produced a progressive loss of flight ability.

The human homolog spectrin repeat-containing nuclear envelope protein 1 (SYNE1) encodes nesprin-1. Mutations in SYNE1 have been identified in patients suffering from spinocerebellar ataxia and autosomal recessive 8 (SCAR8) (Izumi *et al.* 2013), as well as Emery–Dreifuss muscular dystrophy (Zhang *et al.* 2007). It will be of great interest to investigate whether synaptic maintenance is defective in these conditions.

Pumilio is a translational repressor that has functions both in presynaptic motor neurons and postsynaptic muscle (Menon *et al.* 2009). Pum represses expression and translation of the GluR subunit GluRIIA, while mutations in Pum increase levels of GluRIIA dramatically (Menon *et al.* 2004). Our results showing a flight defect upon muscle-specific knockdown suggest that maintaining proper levels of postsynaptic proteins suck as GluRIIA is crucial for maintaining synaptic integrity with age.

The human homolog pumilio RNA-binding family member 2 (PUM2) has been shown to regulate genome stability (Lee *et al.* 2016). Together with our results demonstrated here, this highlights the potential role of maintaining gene expression over time in order to maintain synaptic integrity.

### Intracellular transport

GMAP is uniformly expressed in late embryonic as well as larval stages, where it localizes primarily to the cis side of the Golgi stacks, near the endoplasmic reticulum. Golgi organization and anterograde transport through the Golgi are impaired by overexpression of GMAP (Friggi-Grelin *et al.* 2006). We found a progressive flight defects upon muscle-specific knockdown of GMAP. Thus, it is possible that Golgi sorting and processing are particularly sensitive in the DLMs.

The human homolog, thyroid hormone receptor interactor 11 (TRIP11), similarly encodes a Golgi microtubule-associated protein. Mutations in TRIP11 have been associated with lethal skeletal dysplasia (Smits *et al.* 2010). Specifically, hypomorphic TRIP11 mutations impaired secretion from the Golgi apparatus in patients suffering from odontochondrodysplasia (Wehrle *et al.* 2019).

While Futsch is known to regulate synaptic growth and development as mentioned above, microtubule instability resulting from loss of Futsch also results in defective axonal transport and neurodegeneration (Bettencourt da Cruz *et al.* 2005). Our results showing a progressive loss of flight ability upon knockdown in presynaptic motor neurons suggest that sustained axonal transport is central to synaptic maintenance.

### Intercellular signaling

Our screen also revealed genes associated with intercellular signaling, which is fitting when considering the close connections between neurons, muscles, and glia at these NMJs. *cv-2*, for example, encodes a secreted protein that can either promote or inhibit BMP signaling (Conley *et al.* 2000) and physically binds with the BMP receptor thickveins (Serpe *et al.* 2008). Although the role of cv-2 has been most extensively investigated in wing development, BMP signaling is also critical for synapse development (Aberle *et al.* 2002; Marqués *et al.* 2002; Sweeney and Davis 2002; McCabe *et al.* 2003; Rawson *et al.* 2003; McCabe *et al.* 2004). We also recently identified the need for sustained BMP signaling with age to maintain synaptic integrity using *mayday* mutants (Sidisky *et al.* 2021). Our results here show a requirement of *cv-2* expression within muscles, suggesting a postsynaptic role for maintaining synapses.

Human BMP-binding endothelial cell precursor-derived regulator (BMPER) has also been shown to be a negative regulator of BMP signaling, as BMPER protein inhibits BMP signaling and osteoblast differentiation (Binnerts *et al.* 2004). Additionally, mutations in human BMPER have been associated with the lethal skeletal disorder diaphanospondylodysostosis (Funari *et al.* 2010).

DopR2 is a specific dopamine receptor that is primarily found in the mushroom body, where it plays an important role in olfactory memory (Han *et al.* 1996). DopR2 is also involved in the optomotor response to a moving visual stimulus. Knockdown of DopR2 impairs dopaminergic innervation of the fan-shaped body, impairing this motor response (Akiba *et al.* 2020). Interestingly, knockdown of *DopR2* also results in an upregulation of immune and stress response genes (Regna *et al.* 2016), which is also associated with neurodegeneration (Cao *et al.* 2013).

While these studies have shown a clear role for DopR2 within the *Drosophila* brain, it is surprising that muscle-specific knockdown results in impaired synaptic maintenance. This suggests the possibility that there is dopaminergic innervation in either the indirect flight muscles or some other muscle tissue.

The human gene beta-1-adrenergic receptor (ADRB1) encodes a G-protein-coupled receptor found in greatest abundance in cardiac muscles (Frielle *et al.* 1987), and mutations in ADRB1 are associated with an increased risk of congenital heart failure (Small *et al.* 2002). Although the *Drosophila* homolog functions as a receptor for a different neurotransmitter, our results showing a role for DopR2 in postsynaptic muscle cells suggest that manipulation of neurotransmitter receptor signaling could impact synaptic maintenance.

Overall, our results shown here highlight several genes with wide-ranging functions that are required to maintain synaptic integrity with age. Further mechanistic studies should help further clarify the processes within motor neurons, muscles, and glia that are necessary to maintain these complex structures over time. These results will hopefully serve as a starting point for numerous future studies aimed at revealing neuroprotective mechanisms and strategies for maintaining nervous system function with age.

## Supplementary Material

iyad025_Supplementary_Data

## Data Availability

The authors affirm that the conclusions of the article are fully contained within the article, figures, and tables. All reagents generated in this study will be made available upon request. Supplemental material available at GENETICS online.
