# Supplementary material for: Genome-wide analysis reveals novel regulators of synaptic maintenance in Drosophila
Source: Genetics. 2023 Feb 17;223(4):iyad025. doi: 10.1093/genetics/iyad025 (PMC10078915; doi:10.1093/genetics/iyad025)
Supplement: iyad025_Supplementary_Data [file iyad025_supplementary_data.zip › Figure_S4_GENETICS-2023-305877.pdf]

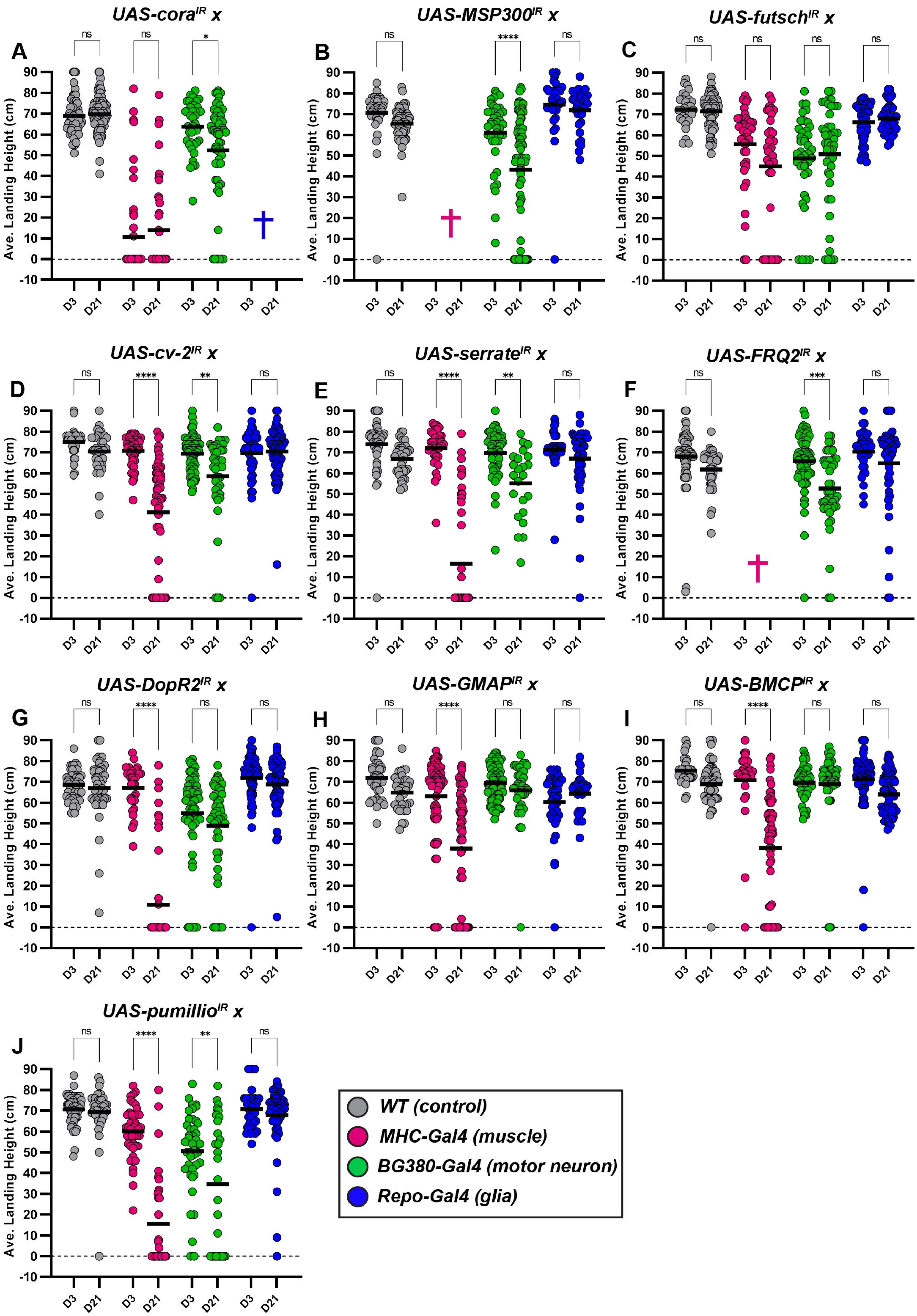

UAS-cora^{IR} x
UAS-MSP300^{IR} x
UAS-futsch^{IR} x
UAS-cv-2^{IR} x
UAS-serrate^{IR} x
UAS-FRQ2^{IR} x
UAS-DopR2^{IR} x
UAS-GMAP^{IR} x
UAS-BMCP^{IR} x
UAS-pumillio^{IR} x
A
B
C
D
E
F
G
H
I
J
Ave. Landing Height (cm)
D3
D21
ns
*
**
***
****
†
WT (control)
MHC-Gal4 (muscle)
BG380-Gal4 (motor neuron)
Repo-Gal4 (glia)
