# Supplementary material for: Genome-wide analysis reveals novel regulators of synaptic maintenance in Drosophila
Source: Genetics. 2023 Feb 17;223(4):iyad025. doi: 10.1093/genetics/iyad025 (PMC10078915; doi:10.1093/genetics/iyad025)
Supplement: iyad025_Supplementary_Data [file iyad025_supplementary_data.zip › Supplemental_Figure_Legends_GENETICS-2023-305877.pdf]

Figure S1. Progressive loss of flight ability linked to synaptic defects.

(A-D) Confocal images of DLM NMJs of DGRP stocks stained with FITC-conjugated HRP (black) at 63X magnification. (E) Quantification of Total Neurite Length (µm). Sample size = n of 10 for each timepoint and genotype. ****p<0.0001; n.s., not significant using a one-way ANOVA with Tukey's Post hoc comparisons. The scale bar in panel D is 20 µm for panels A-D.

Figure S2. Male flight ability in mutants of candidate genes

Measurement of flight ability using males with mutant alleles for each candidate gene. Each allele was assessed in comparison to the appropriate genetic background. (A) Flight ability for mutant alleles with a *white* background. (B) Flight ability for mutant alleles with a *yellow white* background. (C) Flight ability for mutant alleles with an *Oregon-R* background. The average landing height (black bars) for each condition is assessed at Day 3 (gray dots) and Day 21 (purple dots). ****p<0.0001; ***p<0.001; **p<0.01; *p<0.05; n.s., not significant, using one-way ANOVA with Tukey's Post hoc comparisons. Flight ability was analyzed for each condition in triplicate.

Figure S3. mRNA expression level for mutant fly lines. Relative mRNA expression levels (from thorax) of 3 days old female adult flies for the candidate genes A) Coracle, B) MSP300, C) Futsch, D) CV-2, E) Serrate, F) FRQ2, G) GMAP, H) BMCP and I) Pumilio were determined by RT-qPCR. Each mutant line used is described in the respective graph. Except for MSP300 and CV-2, which had two replicates (N=2), each experiment was performed in triplicates (N=3). CV-2 and BMCP were represented by single value (single dot) due to low expression level of both genes; all other values are represented as dots and mean values from replicates measurements are shown as horizontal bars. Wildtype: Oregon R (gray dot); *mutant line*/ +: heterozygous (light blue dot); mutant line (dark glue dot). All experiments were normalized to the reference gene

Actin5C and calibrated to the wildtype. In all panels, p-values were determined using Kruskal–Wallis multiple comparison test: *p< 0.05, **p< 0.01, ns – not significant.

Figure S4. Male flight ability with RNAi knockdown of candidate genes

Measurement of flight ability in males using tissue-specific knockdown of each candidate gene. Each gene was knocked down in muscles (magenta), motor neurons (green), and glia (blue) and also compared to controls (gray). The average landing height (black bars) for each condition is assessed at Day 3 and Day 21. All dots represent individual data points. ****p<0.0001; ***p<0.001; **p<0.01; *p<0.05; n.s., not significant using one-way ANOVA with Tukey's Post hoc comparisons. Flight ability was analyzed for each condition in triplicate.

Figure S5. Flight ability of Gal4 controls.

Measurement of flight ability in females (A) and males (B) with Gal4 drivers alone. Drivers include *MHC-Gal4*, *BG380-Gal4*, and *repo-Gal4.* The average landing height (black bars) for each condition is assessed at Day 3 and Day 21. All dots represent individual data points. **p<0.01; n.s., not significant using one-way ANOVA with Tukey's Post hoc comparisons. Flight ability was analyzed for each condition in triplicate.

Figure S6. mRNA expression level driven by RNAi for the candidate genes. RT-qPCR demonstrate the relative mRNA expression levels in the whole body of flies with knockdown in muscle of A) Coracle, B) MSP300, E) Serrate, F) FRQ2 and I) Pumilio or ubiquitous knockdown of C) Futsch, D) CV-2, G) GMAP, H) BMCP. The knockdown of the candidate genes was driven on muscle and ubiquitously by MHC-gal4 and tubulin-gal4, respectively. mRNA levels were quantified in 3 days old adult female flies' tissues, except for MSP300 and Frq2, where it was utilized 3rd instar larvae. All experiments were performed in triplicates (N=3). Individual values are represented as dots and mean values from replicates measurements are shown as horizontal bars. All experiments were normalized to the reference gene Actin5C and calibrated to the control (control = F1 females or larvae from crossing Oregon-R females and male carrying an RNAi construct for the candidate gene described on each panel). p-values were determined using unpaired t-test: *p< 0.05, **p< 0.01, ***p< 0.001, ns – not significant.

Figure S7. NMJ morphology of Gal4 controls

(A-B) Confocal images of MHC-Gal4 controls at Day 3 and Day 21 stained with FITC-conjugated HRP (black) at 63X magnification. (C) Quantification of Total Neurite Length of MHC-Gal4 controls. (D-E) Confocal images of BG380-Gal4 controls at day 3 and day 21. (F) Quantification of Total Neurite Length of BG380-Gal4 controls. (G-H) Confocal images of repo-Gal4 controls at day 3 and day 21. (I) Quantification of Total Neurite Length of repo-Gal4 controls. Sample size =n of 10 for each timepoint and genotype. ****p<0.0001; ***p<0.001 **p<0.01; *p<0.05 n.s., not significant, using a one-way ANOVA with Tukey's Post hoc comparisons. Each scale bar is 20 µm.

Table S1. Flight scores for each DGRP stock screened.

Average landing heights for each DGRP stock at Day 3 and Day 21. Stocks are sorted and ranked based on the difference between Day 3 and Day 21 values.

Table S2. Top associated annotations provided by the DGRP Portal.

List of the most highly associated SNPs associated with a progressive loss of flight ability. SNP location, major and minor allele frequency, and gene annotations are included.

Table S3: List of primers used in this study.

Annotation of primers used for qPCR analysis of candidate genes.

Table S4: Significance values for RNAi-mediated flight defects

Tabulation of p-values for each comparison made in Figure 10 and Figure S4.
