# Supplementary figures and images for: Genome-wide analysis reveals novel regulators of synaptic maintenance in Drosophila
Source: Genetics. 2023 Feb 17;223(4):iyad025. doi: 10.1093/genetics/iyad025 (PMC10078915; doi:10.1093/genetics/iyad025)

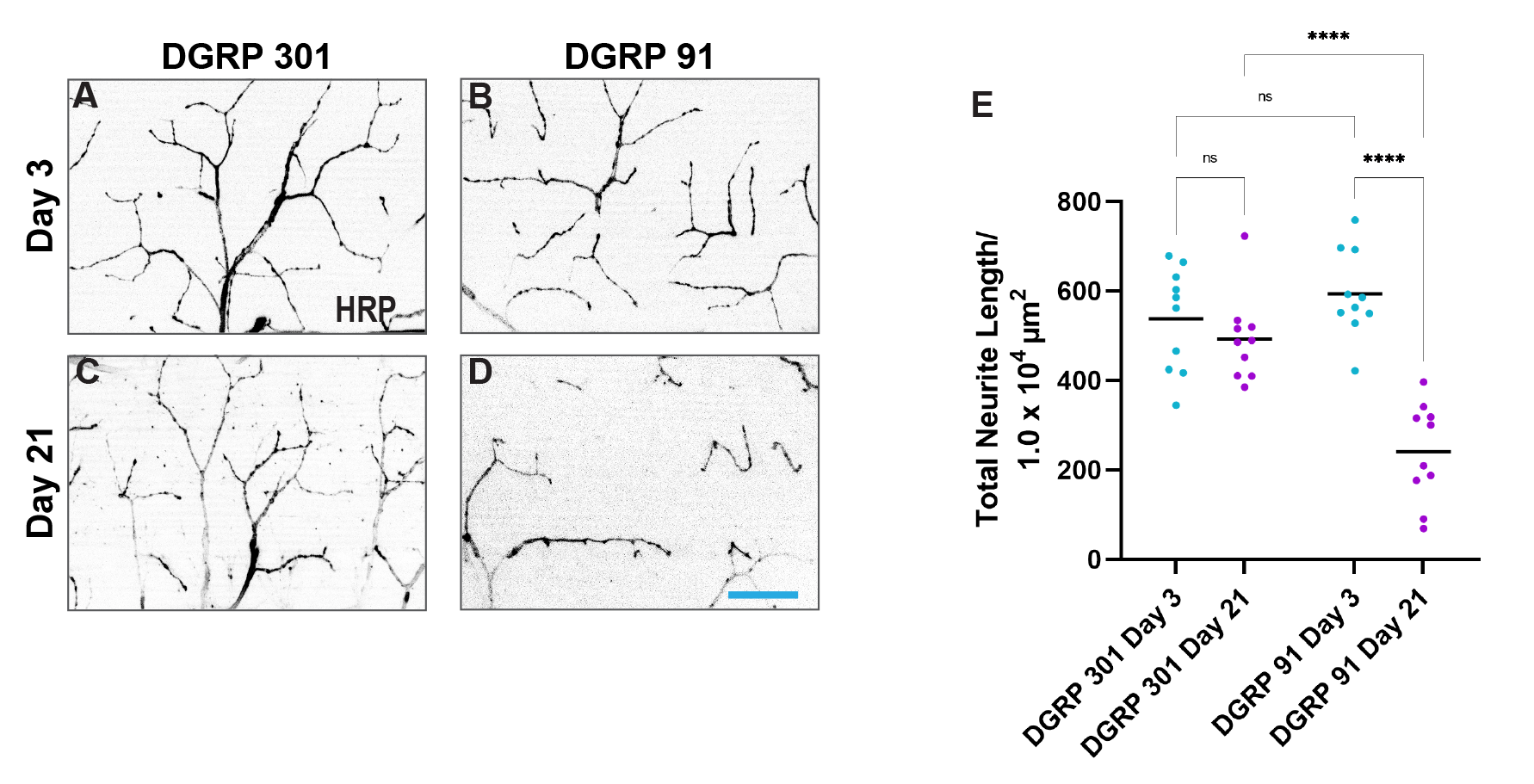

Supplement: iyad025_Supplementary_Data [file iyad025_supplementary_data.zip › Figure_S1_GENETICS-2023-305877.tif]

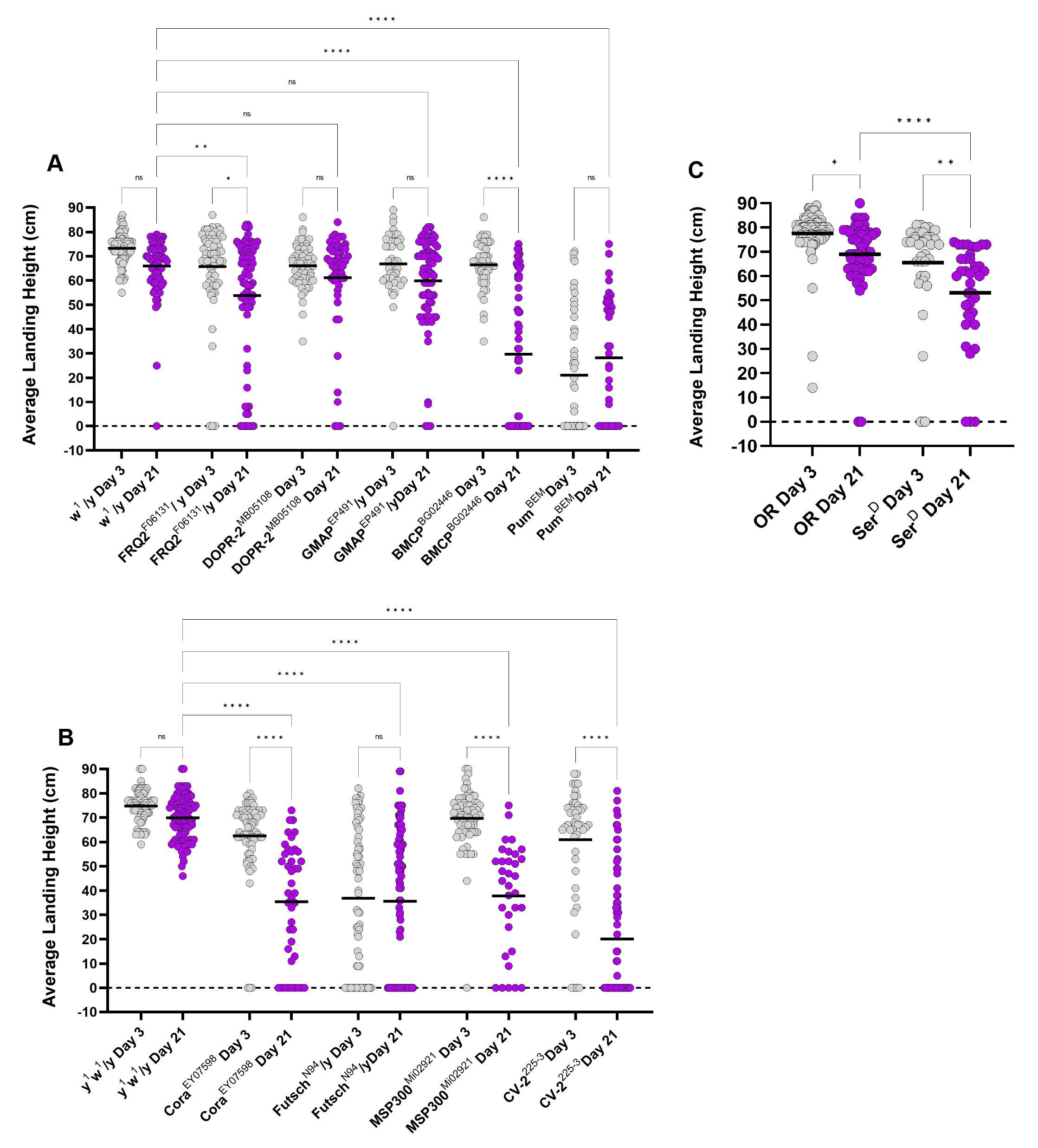

Supplement: iyad025_Supplementary_Data [file iyad025_supplementary_data.zip › Figure_S2_GENETICS-2023-305877.tif]

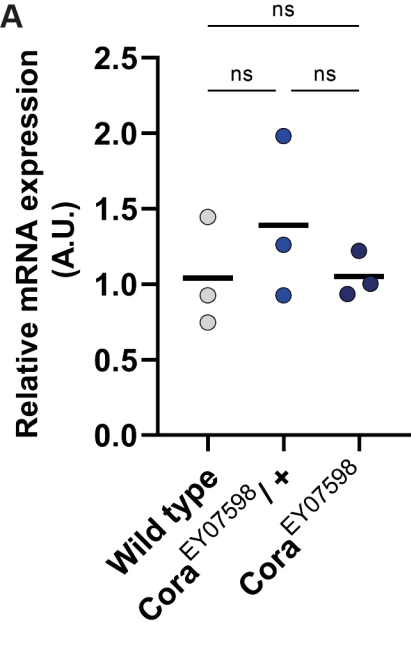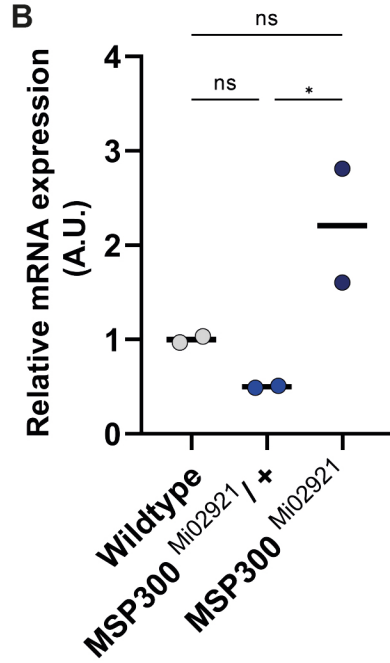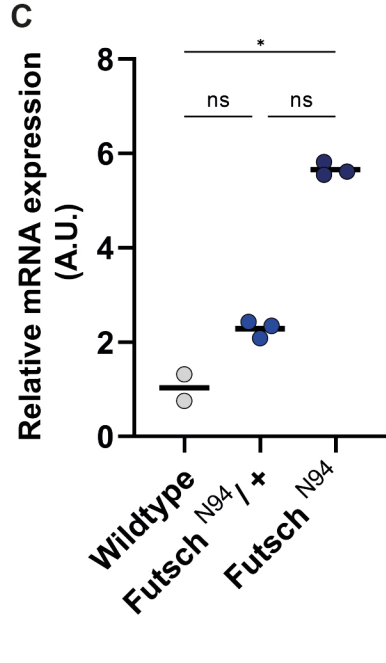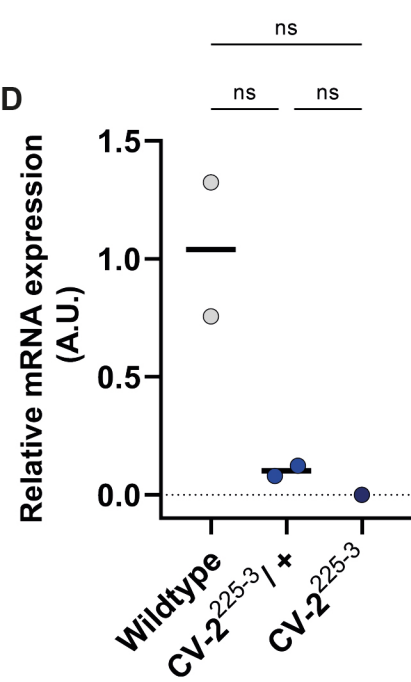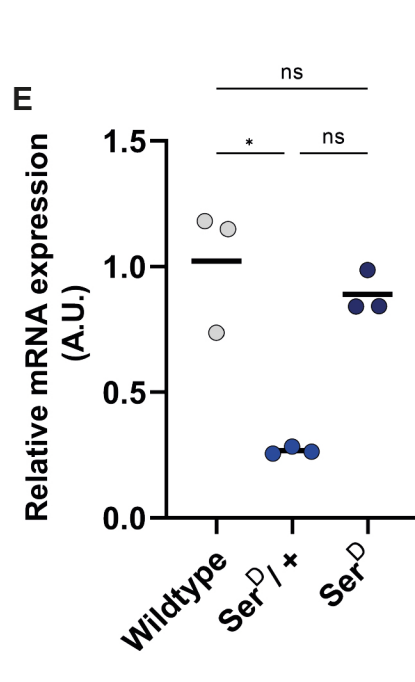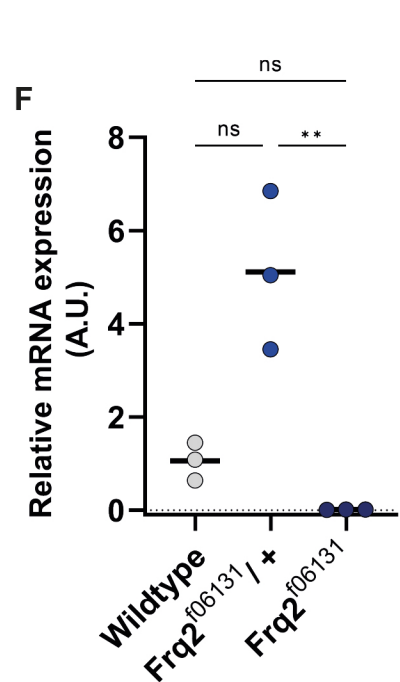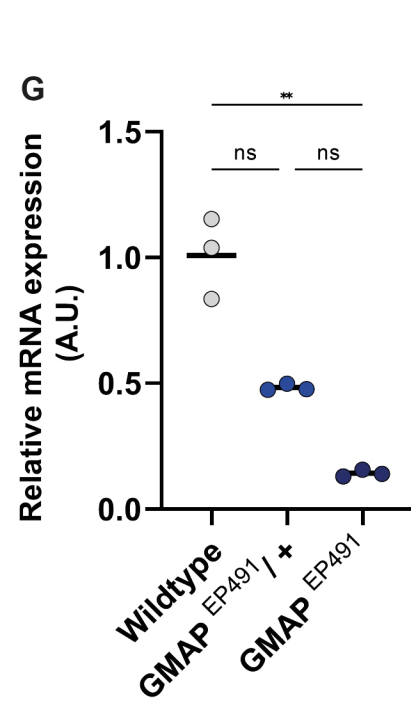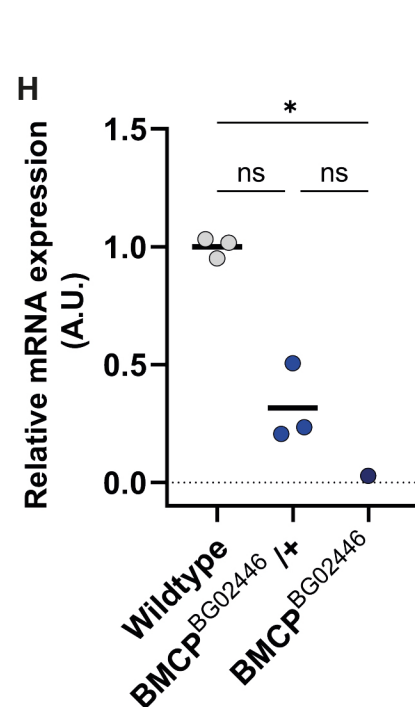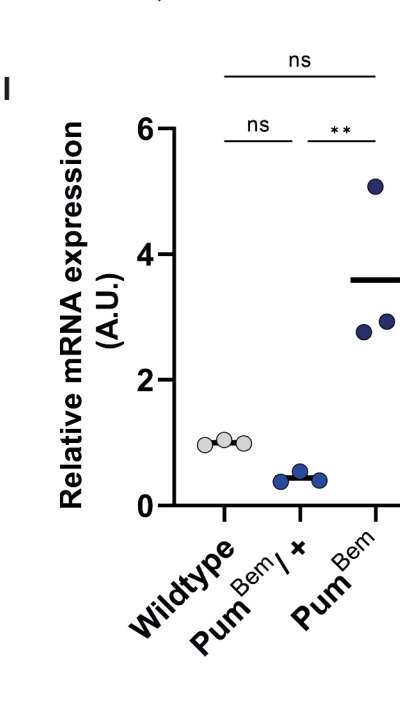

Supplement: iyad025_Supplementary_Data [file iyad025_supplementary_data.zip › Figure_S3_GENETICS-2023-305877.pdf]

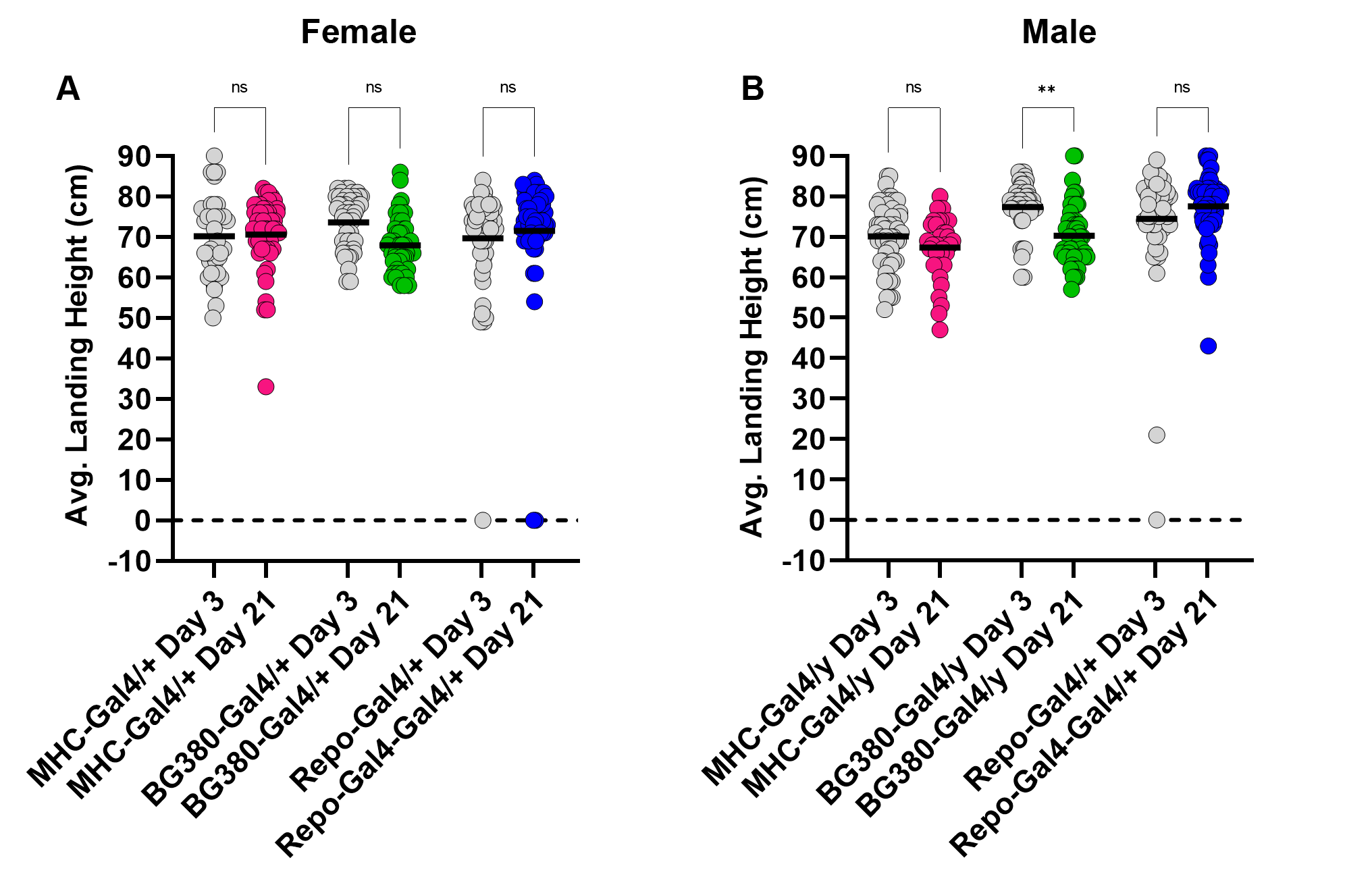

Supplement: iyad025_Supplementary_Data [file iyad025_supplementary_data.zip › Figure_S5_GENETICS-2023-305877.tif]

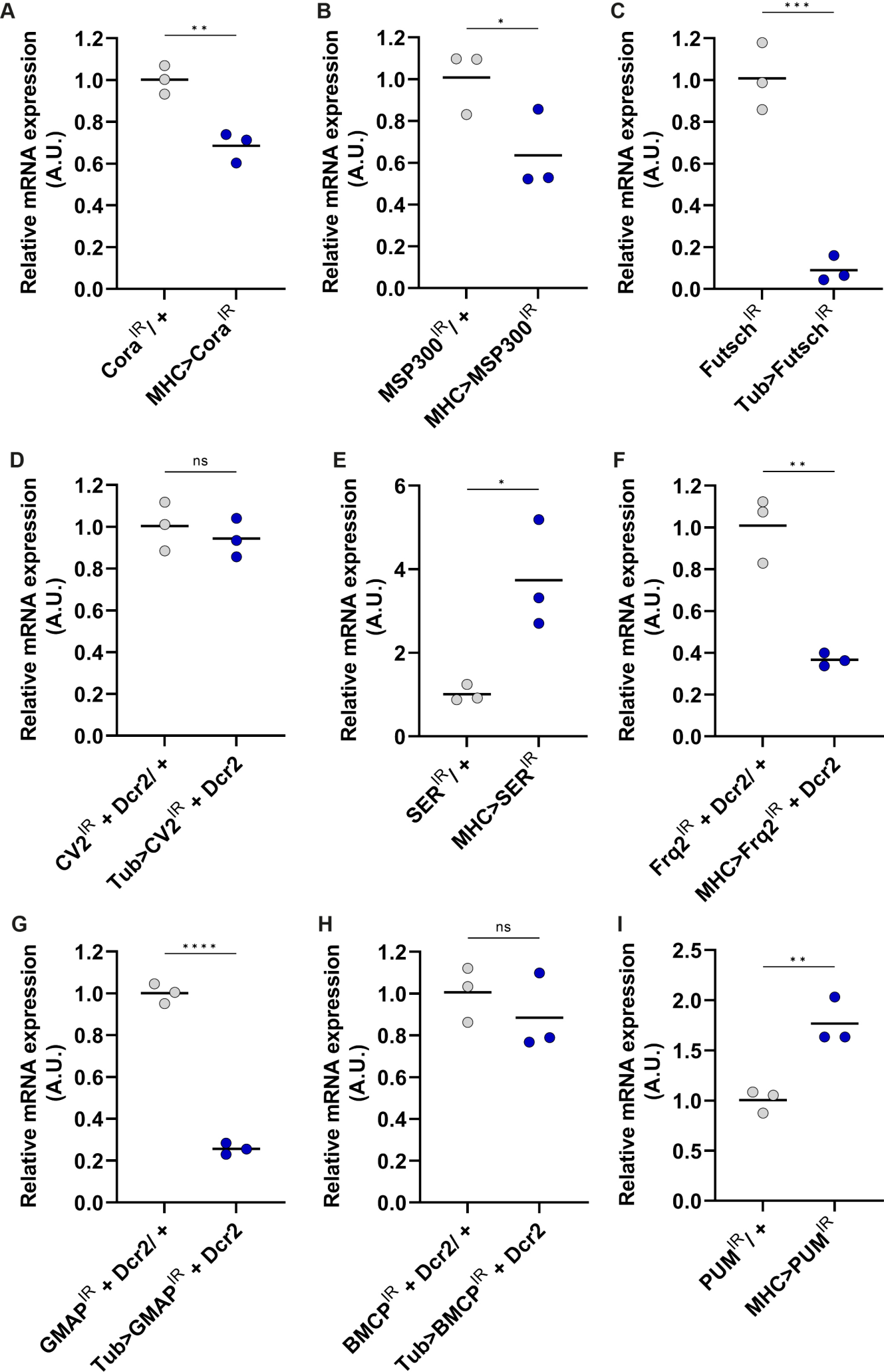

Supplement: iyad025_Supplementary_Data [file iyad025_supplementary_data.zip › Figure_S6_GENETICS-2023-305877.pdf]

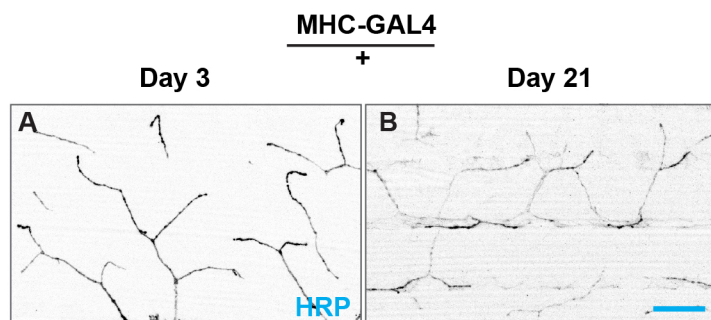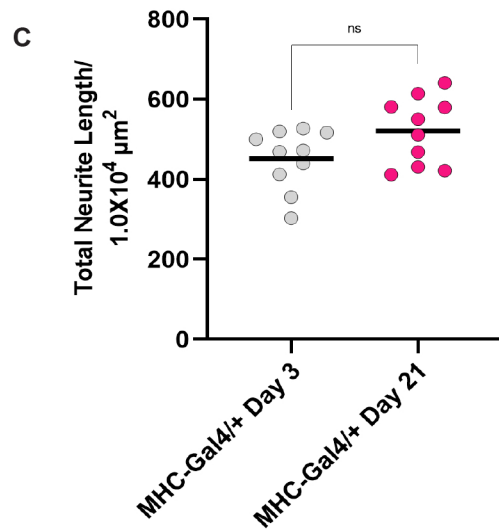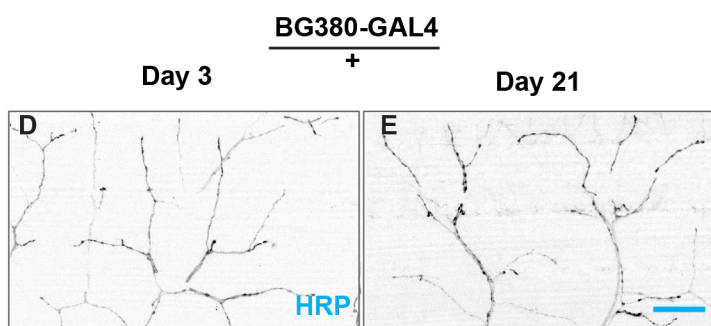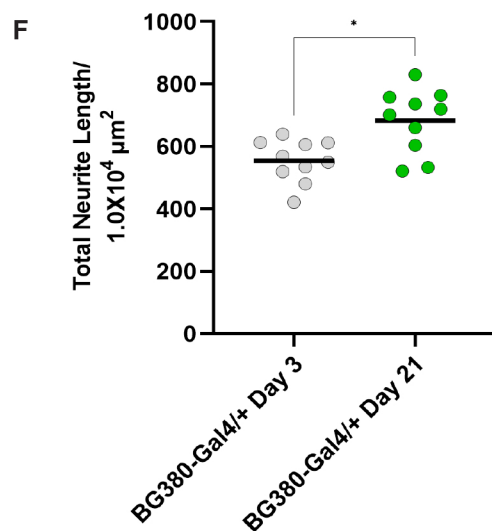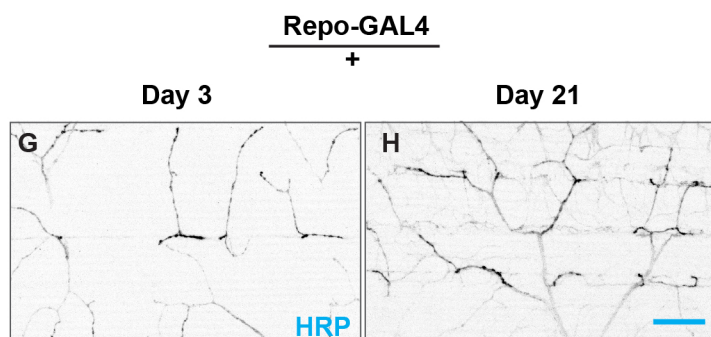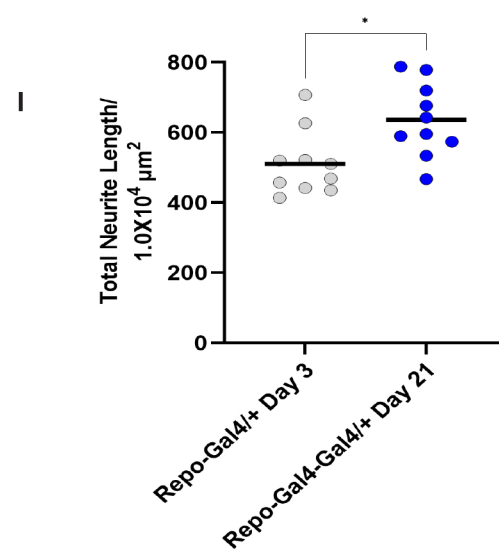

Supplement: iyad025_Supplementary_Data [file iyad025_supplementary_data.zip › Figure_S7_GENETICS-2023-305877.pdf]
